# Design, synthesis, DFT, docking studies and ADME prediction of some new coumarinyl linked pyrazolylthiazoles: Potential standalone or adjuvant antimicrobial agents

**DOI:** 10.1371/journal.pone.0196016

**Published:** 2018-04-19

**Authors:** Sunil Kumar, Vikram Saini, Indresh K. Maurya, Jayant Sindhu, Mukesh Kumari, Ramesh Kataria, Vinod Kumar

**Affiliations:** 1 Department of Chemistry & Centre of Advance Studies in Chemistry, Panjab University, Chandigarhs, India; 2 Department of Biotechnology, AIIMS- New Delhi, Delhi, India; 3 Department of Microbial Biotechnology, Panjab University, Chandigarh, India; 4 K.M. Government College, Narwana, India; 5 Department of Chemistry, Kurukshetra University, Kurukshetra, India; 6 Department of Chemistry, M. M. University, Mullana-Ambala, India; Aligarh Muslim University, INDIA

## Abstract

The control of antimicrobial resistance (AMR) seems to have come to a dead end. The major consequences of the use and abuse of antibacterial drugs are the development of resistant strains due to genetic mutability of both pathogenic and nonpathogenic microorganisms. We, herein, report the synthesis, characterization and biological activities of coumarin-thiazole-pyrazole (CTP) molecular hybrids with an effort to explore and overcome the increasing antimicrobial resistance. The compounds were characterized by analyzing their IR, Mass, ^1^H and^13^C NMR spectral data and elemental analysis. The *in vitro* antimicrobial activity of the synthesized compounds was investigated against various pathogenic strains; the results obtained were further explained with the help of DFT and molecular orbital calculations. Compound **1b** and **1f** displayed good antimicrobial activity and synergistic effects when used with kanamycin and amphotericin B. Furthermore, i*n vitro* cytotoxicity of compounds **1b** and **1f** were studied against HeLa cells (cervical cancer cell) and Hek-293 cells. The results of molecular docking study were used to better rationalize the action and prediction of the binding modes of these compounds.

## Introduction

Over the past few decades, globally antibiotic repository has become less effective due to both antibiotic overuse and an increase in antimicrobial drug resistance. Even common bacterial (e.g. *Staphylococcus aureus*, *Staphylococcus enteric*, *Escherichia coli*) and fungal (e.g. *Candida albicans*, *Cryptococcus neoformans*) pathogens are developing resistance against widely prescribed antibacterial and antifungal drugs like amphotericin B (Amp B), fluconazole (FLC), chloroamphenicol and penicillin [1–3].This is particularly dangerous considering that the major bacterial as well as fungal pathogens such as *Candida* sp. account for ~75% of all infections and represent the 4^th^ leading cause of nosocomial infections [4]. Thus microbial drug resistance has emerged as a major public health challenge across the world. Consequently, strategies are needed to develop new therapeutic compounds that could either act as new drugs or could complement the existing drug therapy more effective by acting as an adjuvant [5–7].

**Fig 1:**
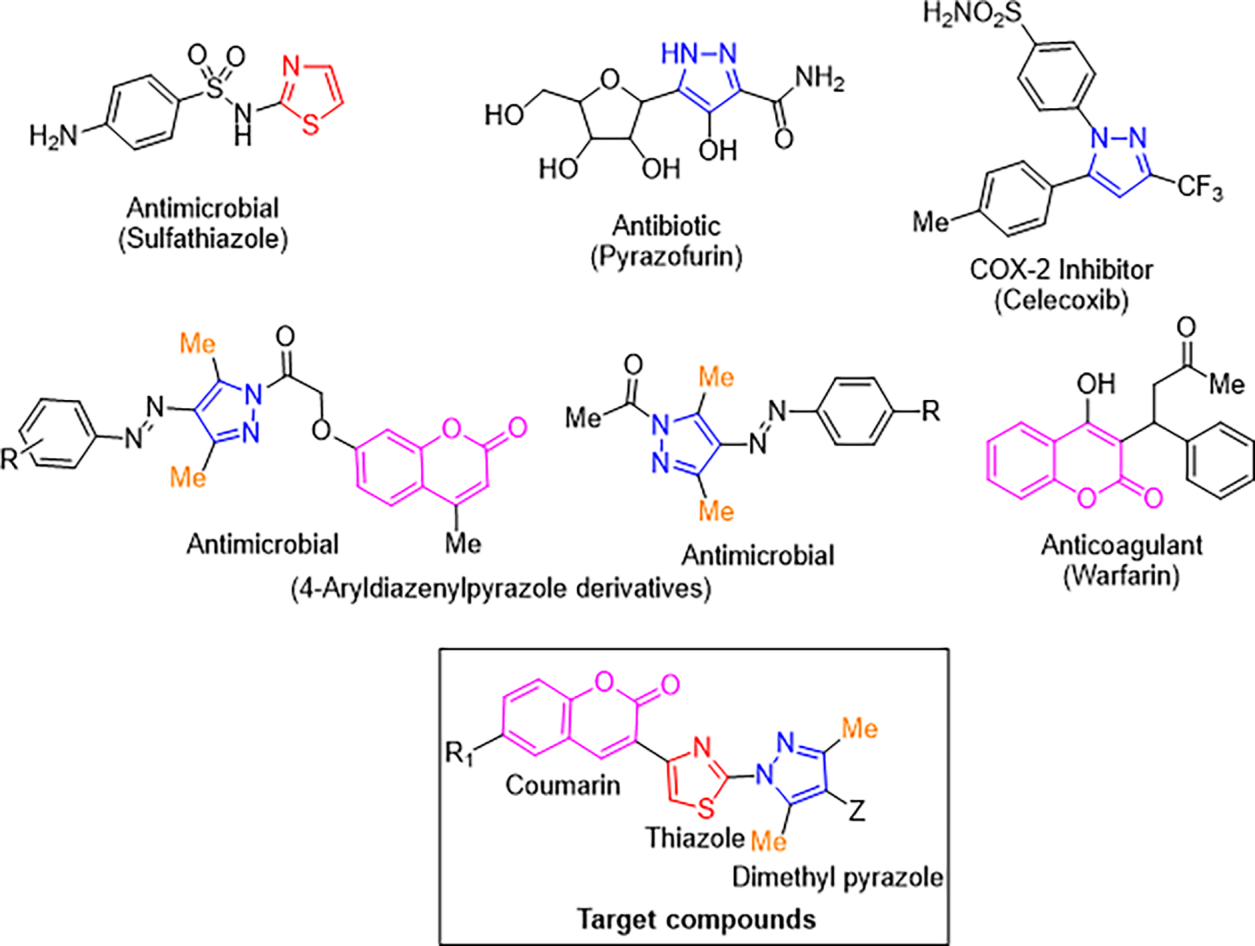
Some thiazole, pyrazole, 4-aryldiazenylpyrazole and coumarin containing biological active heterocycles and target compounds.

In recent past, azoles have garnered considerable attention especially in the design and synthesis of compounds with significant biological activities [8–11]. In particular, there are various drugs incorporating thiazole nucleus (Fig 1) which are being used as the antimicrobial (sulfathiazole), antiinflammatory (fanetizole) and antifungal (abafungin) agents [12–17]. Similarly, compounds containing pyrazole ring have been found to exhibit a variety of biological properties and many of them such as celecoxib, pyrazofurin etc. (Fig 1) are used clinically besides using for agricultural pest management [18–22]. Especially, 3,5-dimethyl-4-aryldiazenylpyrazole derivatives have been reported to exhibit cytotoxic [23], antioxidant [24], analgesic [25] and significant antimicrobial potential [26]. On the other side, compounds containing coumarin moiety (Fig 1) have also been proved as a potent antimicrobial, antitumor, anticoagulant, antiviral, and anti-inflammatory agents [23,24,27–31]. It has been widely accepted that structural properties of thiazole, pyrazole and coumarin derivatives and their stability under *in vivo* conditions are the primary factors for their superior pharmacological activities [32–34].

A drug discovery program involves the planning of novel chemical entities using molecular hybridization approach where two active pharmacophoric units of known drugs were connected through a covalent bond in a single matrix [35–37]. The selection of the active units in the dual drug is usually established on their observed (or anticipated) synergistic or additive pharmacological activities. In prospect of the above discussed facts and in continuation of our research towards the development of novel molecular hybrids as potential biologically active agents [38–42], we have decided to build new molecular hybrids based on coumarin-thiazole-pyrazole (CTP).

## Experimental

### Chemistry

#### General

Melting points were determined by open capillary method and are uncorrected. Infrared spectrum was obtained neat on a Thermo Scientific Fischer spectrometer. Multinuclear NMR (^1^H, ^13^C) spectra were recorded on a Bruker avance at 300 MHz. Chemical shifts are reported in parts per million (ppm). Tetramethylsilane served as an internal standard in ^13^C and ^1^H NMR (δ 0.00 ppm). CHNS analyses were obtained on a Perkin Elmer Model 2400 CHNS elemental analyzer and Mass spectroscopy data of synthesized compound was recorded on waters QQ-TOF micro Mass Spectrometer. All the chemicals used for the synthesis of target compounds have been purchased from Sigma Aldrich and were used as received. All the compounds gave C, H and N analysis within ± 0.5 of the theoretical values. Scanned Spectra for all compounds are shown in S3–S19 Figs.

#### General procedure for the synthesis of 4-(coumarin-3-yl)-2-((3,5-dimethyl-4-phenyldiazenyl)pyrazol-1-yl)thiazoles (1a-1g)

An ethanolic solution (20 mL) of 3-bromoacetylcoumarin (**7**, 0.52 g, 2.00 mmol), sodium carbonate (0.11 g, 1.00 mmol) and 3,5-dimethyl-4-(*p*-chlorophenyl)diazenylpyrazole-1-thiocarboxamide (**5a) (**0.59 g, 2.00 mmol) was refluxed on a water bath for 6 h. On completion of the reaction as observed using TLC (20:80, Ethylacetate: Hexane (*v/v*)), the solvent was evaporated. The solid thus obtained was dissolved in chloroform (25 mL) and washed with water (50 mL). The organic layer was separated and the excess solvent was distilled off. The solid thus obtained was crystallized with ethanol to afford **1a** in 82% yield. The protocol was further extended using differently substituted amines and coumarin which resulted into the formation of compounds **1b-1g** in good to excellent yield.

### Spectral data

**4-(Coumarin-3-yl)-2-(3,5-dimethyl-4-(4-chlorophenyl)diazenyl)pyrazol-1-yl)thiazole (1a)**. R_f_: 0.6; mp 214–216°C; Yield: 82%; IR (cm^-1^): 1719 (C = O), 1605 (C = N); ^1^H NMR (300 MHz, CDCl_3_, δ ppm): 8.39 (s, 1H, coumarin 4-H), 8.06 (s, 1H, thiazole 5-H), 7.70 (d, 2H, *J* = 8.4 Hz, *p*-chlorophenyl 2, 6-H), 7.65 (s, 1H, coumarin 5-H), 7.60–7.52 (m, 1H, coumarin 7-H), 7.45 (d, 2H, *J* = 8.4 Hz, *p*-chlorophenyl 3,5-H), 7.41–7.33 (m, 2H, coumarin 6,8-H), 2.86 (s, 3H), 2.31 (s, 3H); ^13^C NMR (75 MHz, CDCl_3_, δ ppm): 164.33 (C = O), 159.22 (C_2_-thiazole), 154.38 (C_8'_-coumarin), 149.26 (C_4_-thiazole), 147.14 (C_4_-coumarin), 141.59 (C-Ar), 136.64 (C-Ar), 133.14 (C-Ar), 129.23 (C-Ar), 128.82 (C-Ar), 125.07 (C-Ar), 123.35 (C-Ar), 120.87 (C-Ar), 119.40 (C-Ar), 118.67 (C-Ar), 116.81 (C-Ar), 110.77 (C-Ar), 23.88 (5-CH_3_ pyrazole), 14.70 (3-CH_3_ pyrazole); MS (m/z) Found: 463.1, 461.0 (3:1) [M+H]^+^; Calcd.: 461.92;Anal. Calcd. for C_23_H_16_ClN_5_O_2_S: C, 59.80; H, 3.49; N, 15.16. Found: C, 59.85; H, 3.50; N, 15.46.

**4-(6-Chlorocoumarin-3-yl)-2-(3,5-dimethyl-4-(4-bromophenyl)diazenyl)pyrazol-1-yl) thiazole (1b).** R_f_: 0.6; mp 220–222°C;Yield: 88%; IR (cm^-1^): 1719 (C = O), 1602 (C = N); ^1^H NMR (300 MHz, CDCl_3_, δ): 8.30 (s, 1H, coumarin 4-H), 8.08 (s, 1H, thiazole 5-H), 7.65–7.61 (m, 5H, coumarin 5-H, Ph2,6,3,5-H), 7.49 (d, 1H, *J* = 8.7 Hz, coumarin 8-H), 7.31 (d, 1H, *J* = 8.7 Hz, coumarin 7-H), 2.74 (s, 3H, 5-CH_3_), 2.20 (s, 3H, 3-CH_3_); ^13^C NMR (75 MHz, CDCl_3_, δ ppm): 167.47 (C = O), 159.23 (C_2_-thiazole), 154.49 (C_8'_-coumarin), 149.56 (C_4_-thiazole), 147.15 (C_4_-coumarin), 143.40 (C-Ar), 137.33 (C-Ar), 133.24 (C-Ar), 128.86 (C-Ar), 125.07 (C-Ar), 124.26 (C-Ar), 124.16 (C-Ar), 122.74 (C-Ar), 119.53 (C-Ar), 118.64 (C-Ar), 116.89 (C-Ar), 115.04 (C-Ar), 112.54 (C-Ar), 31.16 (5-CH_3_ pyrazole), 25.30 (3-CH_3_ pyrazole); MS (m/z) Found: 542.1, 540.1; Calcd.: 540.82; Anal. Calcd. for C_23_H_15_BrClN_5_O_2_S: C, 51.08; H, 2.80; N, 12.95. Found: C, 51.00; H, 2.82; N, 13.14.

**4-(Coumarin-3-yl)-2-(3,5-dimethyl-4-(4-methylphenyl)diazenylpyrazol-1-yl)thiazole (1c).** R_f_: 0.6; mp 240–241°C; Yield: 80%; IR (cm^-1^): 1713 (C = O), 1601 (C = N); ^1^H NMR (300 MHz, CDCl_3_, δ): 8.32 (s, 1H, coumarin 4-H), 8.02 (s, 1H, thiazole 5-H), 7.87 (d, 2H, *J* = 8.7 Hz, Ph 2,6-H), 7.71 (d, 1H, *J* = 8.4 Hz, coumarin 5-H), 7.49 (d, 2H, *J* = 8.4 Hz, Ph 3,5-H), 7.62 (t, 1H, *J* = 7.5 Hz, coumarin 7-H), 7.43–7.40 (m, 2H, coumarin 6,8-H), 2.74 (s, 3H, 5-CH_3_), 2.20 (s, 3H, 3-CH_3_), 2.05 (s, 3H, *p*-CH_3_); ^13^C NMR (75 MHz, CDCl_3_, δ ppm): 162.73 (C = O), 157.90 (C_2_-thiazole), 147.90 (C_8'_-coumarin), 140.92 (C_4_-thiazole), 140.62 (C_4_-coumarin), 139.09 (C-Ar), 134.47 (C-Ar), 133.02 (C-Ar), 132.82 (C-Ar), 130.27 (C-Ar), 128.85 (C-Ar), 128.44 (C-Ar), 128.39 (C-Ar), 128.35 (C-Ar), 126.95 (C-Ar), 126.92 (C-Ar), 125.36 (C-Ar), 123.28 (C-Ar), 118.07 (C-Ar), 117.99 (C-Ar), 113.57 (C-Ar), 35.10 (5-CH_3_ pyrazole), 29.61 (3-CH_3_ pyrazole), 14.60 (Ph-CH_3_); MS (m/z) found: 441.3; Calcd. 441.50; Anal. Calcd. For C_24_H_19_N_5_O_2_S: C, 65.29; H, 4.34; N, 15.86; Found C, 65.69; H, 4.26; N, 15.85.

**4-(6-Chlorocoumarin-3-yl)-2-(3,5-dimethyl-4-(4-methylphenyl)diazenyl)pyrazol-1-yl) thiazole (1d).** R_f_: 0.6; mp 255–256°C; Yield: 83%; IR (cm^-1^): 1720 (C = O), 1601 (C = N); ^1^H NMR (300 MHz, CDCl_3_, δ): 8.39(s, 1H, coumarin 4-H), 8.00(s, 1H, thiazole 5-H), 7.86 (d, 2H, *J* = 8.4 Hz, Ph2,6-H), 7.66 (s, 1H, coumarin 5-H), 7.63 (d, 2H, *J* = 8.1 Hz, Ph3,5-H), 7.53 (d, 1H, *J* = 8.4 Hz, coumarin 7-H), 7.35 (d, 1H, *J* = 8.4 Hz, coumarin 8-H), 2.69 (s, 3H, 5-CH_3_), 2.19 (s, 3H, 3-CH_3_), 1.98 (s, 3H, *p*-CH_3_);^13^C NMR (75 MHz, CDCl_3_, δ ppm): 164.37 (C = O), 152.86 (C_2_-thiazole), 151.56 (C_8'_-coumarin), 144.30 (C_4_-thiazole), 143.71 (C_4_-coumarin), 139.30 (C-Ar), 138.86 (C-Ar), 131.69 (C-Ar), 131.60 (C-Ar), 129.69 (C-Ar), 128.69 (C-Ar), 128.41 (C-Ar), 126.37 (C-Ar), 126.20 (C-Ar), 120.61 (C-Ar), 120.24 (C-Ar), 119.41 (C-Ar), 116.44 (C-Ar), 113.44 (C-Ar), 20.94 (CH_3_-Ph), 24.37 (5-CH_3_ pyrazole), 13.86 (3-CH_3_ pyrazole); MS (m/z) Found: 477.6, 475.8; Calcd. 475.95; Anal. Calcd. for C_24_H_18_ClN_5_O_2_S: C, 60.56; H, 3.81; N, 14.71; Found C, 60.55; H, 3.71; N, 14.63.

**4-(Coumarin-3-yl)-2-(3,5-dimethyl-4-(4-bromophenyl)diazenyl)pyrazol-1-yl)thiazole (1e).** R_f_: 0.6; mp 210–212°C; Yield: 88%; IR (cm^-1^): 1717 (C = O), 1600 (C = N); ^1^H NMR (300 MHz, CDCl_3_, δ): 8.30 (s, 1H, coumarin 4-H), 8.08 (s, 1H, thiazole 5-H), 7.82 (d, 1H, *J* = 8.1 Hz, coumarin 5-H), 7.71 (d, 2H, *J* = 8.4 Hz, Ph 3,5-H), 7.65–7.61 (m, 1H, coumarin 7-H), 7.47 (d, 2H, *J* = 8.4 Hz, Ph2,6-H), 7.28–7.24 (m, 2H, coumarin 6,8-H), 2.72 (s, 3H, 5-CH_3_), 2.32 (s, 3H, 3-CH_3_);^13^C NMR (75 MHz, CDCl_3_, δ ppm): 164.81 (C = O), 159.22 (C_2_-thiazole), 154.38 (C_8'_-coumarin), 149.26 (C_4_-thiazole), 147.14 (C_4_-coumarin), 141.59 (C-Ar), 136.64 (C-Ar), 133.14 (C-Ar), 129.23 (C-Ar), 128.82 (C-Ar), 125.07 (C-Ar), 123.35 (C-Ar), 121.87 (C-Ar), 119.40 (C-Ar), 118.67 (C-Ar), 116.81 (C-Ar), 110.77 (C-Ar), 25.08 (5-CH_3_ pyrazole), 14.71 (3-CH_3_ pyrazole); MS (m/z) found: 506.1, 504.1 (3:1); Calcd.: 506.37; Anal. Calcd. for C_23_H_16_BrN_5_O_2_S: C, 54.55; H, 3.18; N, 13.83. Found: C, 54.48; H, 3.12; N, 13.82.

**4-(6-Chlorocoumarin-3-yl)-2-(3,5-dimethyl-4-(4-chlorophenyl)diazenyl)pyrazol-1-yl) thiazole (1f).** R_f_: 0.6; mp 235–236°C;Yield: 85%; IR (cm^-1^): 1719 (C = O), 1605 (C = N); ^1^H NMR (300 MHz, CDCl_3_, δ): 8.28 (s, 1H, coumarin 4-H), 8.07 (s, 1H,thiazole 5-H), 7.79 (d, 1H, *J* = 2.4 Hz, coumarin 5-H), 7.65–7.61 (m, 5H, Ph 4-H and coumarin 6-H), 7.28 (d, 1H, *J* = 8.1 Hz, coumarin 8-H), 2.84 (s, 3H, 5-CH_3_), 2.21 (s, 3H, 3-CH_3_); ^13^C NMR (75 MHz, CDCl_3_, δ ppm 167.44 (C = O), 159.23 (C_2_-thiazole), 150.22 (C_8'_-coumarin), 147.40 (C_4_-thiazole), 142.56 (C_4_-coumarin), 139.21 (C-Ar), 133.23 (C-Ar), 128.88 (C-Ar), 125.10 (C-Ar), 124.85 (C-Ar), 124.80 (C-Ar), 122.43 (C-Ar), 119.34 (C-Ar), 118.64 (C-Ar), 116.81 (C-Ar), 113.49 (C-Ar), 110.39 (C-Ar), 25.16 (5-CH_3_ pyrazole), 15.54 (3-CH_3_ pyrazole); MS (m/z) found: 497.2, 495.2 (3:1); Calcd.: 496.37; Anal. Calcd. for C_23_H_15_Cl_2_N_5_O_2_S: C, 55.65; H, 3.05; N, 14.11; Found: C, 55.64; H, 3.10; N, 14.17.

**4-(Coumarin-3-yl)-2-(3,5-dimethyl-4-phenyldiazenyl)pyrazol-1-yl)thiazole (1g).** R_f_: 0.6; mp 205–206°C; Yield: 82%; IR (cm^-1^): 1715 (C = O), 1603 (C = N); ^1^H NMR (300 MHz, CDCl_3_, δ): 8.34 (s, 1H, coumarin 4-H), 8.08 (s, 1H, thiazole 5-H), 7.82–7.78 (m, 2H, Ph 2,6-H), 7.78 (d, 1H, *J* = 8.4 Hz, coumarin 5-H), 7.65–7.63 (m, 1H, coumarin 7-H), 7.62–7.57 (m, 4H, Ph 3,4,5-H, coumarin 6-H), 7.33 (d, 1H, *J* = 8.7 Hz, coumarin 8-H), 2.79 (s, 3H, 5-CH_3_), 2.24 (s, 3H, 3-CH_3_); ^13^C NMR (75 MHz, CDCl_3_, δ ppm): 167.79 (C = O), 159.35 (C_8'_-coumarin), 154.35 (C_2_-thiazole), 148.40 (C_4_-coumarin), 146.95 (C_4_-thiazole), 142.99 (C-Ar), 138.95 (C-Ar), 135.97 (C-Ar), 132.95 (C-Ar), 128.78 (C-Ar), 124.99 (C-Ar), 123.43 (C-Ar), 122.59 (C-Ar), 120.05 (C-Ar), 119.79 (C-Ar), 118.76 (C-Ar), 116.75 (C-Ar), 110.52 (C-Ar), 24.83 (5-CH_3_ pyrazole), 15.22 (3-CH_3_ pyrazole); MS (m/z) found: 427.3;Calcd.: 427.28; Anal. Calcd.forC_23_H_17_N_5_O_2_S: C, 64.62; H, 4.01; N, 16.38; Found C, 64.55; H, 4.05; N, 16.45.

### Antimicrobial assay

The microbial strains (bacterial and fungal) used in the study were obtained from the Institute of Microbial Technology (MTCC-IMTECH), Chandigarh and National Collection of Pathogenic Fungi (NCPF), Post-Graduate Institute of Medical Education and Research (PGIMER), Chandigarh. The bacterial strains *Escherichia coli MTCC 2961*, *Staphylococcus aureus MTCC 3160*, *Enterococcus faecalis MTCC 439*, *Vibrio cholera MTCC 3906*, *Streptococcus pyogenes MTCC 442* were cultured in Mullar Hinton broth (MHB, HiMedia, India). The fungal strains *Candida albicans NCPF 400034*, *Candida glabrata MTCC3019*, *Candida krusei NCPF 44002*, *Candida parapsilosis NCPF 450002*, *Candida keyfer*, *NPCPF 410004 and Candida tropicalis NPCPF420007* were cultured in yeast extract-peptone-dextrose (YEPD broth, HiMedia, India) and RPMI 1640 media (HiMedia, India). For agar plates, 2.5% (w/v) bacteriological agar (HiMedia, India) was added to the medium. The strains were stored with 15% glycerol at -80°C as frozen stocks. The cells were freshly revived on respective agar plates from the stock before each experiment [43,44].

### Antibiotic susceptibility testing

#### Antibacterial activity

All the bacterial strains (*E*. *coli*, *S*. *aureus*, *E*. *faecalis*, *V*. *cholerae*, *S*. *pyogenes*) were grown overnight and were diluted in Mueller-Hinton broth (MHB) to a cell density of 10^5^ CFU/mL. 100 μL of this culture and compounds **1a-1g** (250–0.112 μM) dissolved in DMSO, were added into the 96-well flat bottomed microtiter plate (Genexy-HiMedia, India). The plate was incubated at 37°C without shaking for 24 h. The visual and optical density was measured using microplate reader at 600 nm (Thermo, Model 680). The minimum inhibitory concentration (MIC) was defined as the concentration of the antimicrobial agent that inhibit >99% growth. kanamycin (KAN), a known antibacterial drug is used as a positive control [45].

#### Antifungal activity

The antifungal activities of compounds **1a-1g** against fungal species (*C*. *albicans*, *C*. *glabrata*, *C*. *krusei*, *C*. *parapsilosis*, *C*. *keyfer*, *C*. *tropicalis*) were performed according to the Clinical and Laboratory Standards Institute (CLSI, formerly NCCL) in RPMI-1640 medium by broth micro-dilution methods [46]. The concentrations of compounds **1a-1g** ranged between 250 μM and 0.097 μM. The 96 well flat bottom microtiter plates were incubated without shaking at 30°C for 48 h. The visual and optical density was measured using microplate reader at 492 nm (Thermo, Model 680) to study the inhibition in fungal growth. The MIC was defined as the lowest concentration of antifungal drug which resulted in >99% inhibition of growth compared to that of the untreated control. amphotericin B, a known antifungal drug is used as a positive control.

#### Time kill assay

*S*. *aureus* exponential phase cells (~1 x 10^5^ CFU/mL) was inoculated in MHB medium containing compound **1f** (at 15.67 μM conc.). The *S*. *aureus* cells were incubated (37°C; 200 rpm) and 100 μl aliquots were removed at pre-determined time points (0, 4, 8, 12, 16, 20 and 24 h). The aliquots were serially diluted (10 fold) in saline and plated on the MHA (Muller Hinton Agar) plates. The numbers of colonies were counted after incubating the plates at 37°C for 24 h. Similarly *C*. *keyfer* exponential phase cells (~1 x 10^4^ CFU/mL) were inoculated in RPMI 1640 medium containing compound **1f** (62.25 μM). The tubes were incubated (30°C; 200 rpm), and 100 μL aliquots were removed at pre-determined time points (0, 4, 8, 12, 16, and 24 h). The aliquots were serially diluted (10 fold) in saline water and plated on YEPD agar plates. The numbers of colonies were counted after incubating the plates at 30°C for 48 h [44].

#### Checkerboard assay of compound 1b and 1f

The interaction of compound **1b** and **1f** with well-known antimicrobials kanamycin, and amphotericin B and fluconazole were evaluated by the checkerboard method as described previously and represented as the fractional inhibitory concentration (FIC) index (FICI) i.e. sum of the FIC for each compounds. FIC value of the most effective combination was used in calculating the FICI. FICI = FIC of X + FIC of Y = C_X_^comb^/ MIC_X_^alone^+ C_X_^comb^/ MIC_Y_^alone^, Where, MIC_X_^alone^ and MIC_Y_^alone^ are the MICs of drug X and Y when acting alone and C_X_^comb^ and C_Y_^comb^ are concentrations of drugs X and Y at the isoeffective combinations, respectively. The interaction was described as synergistic when the FICI was ≤ 0.5, and when > 4.0, it was interpreted as antagonist and any value in between as in different [45].

#### Scanning electron microscopy (SEM) of 1f treated *S*. *aureus* and *C*. *keyfer* cells

The cell suspensions of *S*. *aureus* and *C*. *keyfer* in exponential phase were prepared in MHB and RPMI 1640 medium (pH 7), respectively. Compound **1f** (15.67μM) was added to the *S*. *aureus* cells (~1 x 10^5^ CFU/mL), whereas **1f** (62.25 μM) was added to the *C*. *keyfer* (~1 x 10^4^ CFU/mL) and incubated at 37°C (*S*. *aureus* for 12 h) and 30°C (*C*. *keyfer* for 12 h). After treatment the samples for SEM study were prepared as described previously and observed under the SEM (JEOL-6100F, JEOL, Japan) [47].

#### Mammalian cell toxicity

The mammalian toxicity of compounds **1a-1g** against Hek-293 (normal human embryonic kidney cells) and HeLa (human cervical cancer cells) by MTT (3-(4,5-dimethylthiazol-2-yl)-2,5-diphenyltetrazolium bromide) protocol [48]. Briefly, the cells (5 × 10^4^ /well) were cultured in RPMI 1640 medium supplemented with 10% Fetal Bovin Serum (FBS) in 96-well microtiter plate at 37°C for overnight. The next day compounds **1a**-**1g** at MIC conc. were added to the cells in separate wells and incubated at 37°C for 18 h. The cells were further incubated at 37°C for 3 h to 4 h in 20 μL of MTT solution (5 mg/mL) in phosphate buffer saline (PBS). Further, after removing supernatant (120 μL), Dimethyl sulfoxide (DMSO; 100 μL) was added, and the suspension was mixed to dissolve the formazan crystals. The percent viability of cells was calculated by the ratio of O.D_570_ of treated cells to the O.D_570_ of untreated cells. Untreated cells and novobiocin (aminocoumarin antibiotic) were taken as negative and positive control, respectively.

### Computational methods

#### DFT studies

Khon-Sham’s DFT method that undergo the gradient-corrected hybrid density functional B3LYP were used for all theoretical calculations [49]. The Becke’s three parameters functional of Lee et al [50], is a combination of non-local exchange potential with the non-local correlation function. A full geometry optimization was performed for all structures, using this function [50] and the 6–31+g(d,p) bases set as implemented by Gaussian 03 package [51]. All geometries were visualized using Avogadro 1.2 software package.

#### Molecular docking

The protein-ligand crystal structure has been optimized to its lower energy conformation using protein preparation wizard where all the water molecules were removed during preprocess and missing side chains of residues have been added using prime. Keeping in mind the appropriate ionization states for the acidic as well as basic amino acids, hydrogen atoms were added to the protein structure corresponding to the physiological pH 7.0. Finally energy minimization with root-mean-square deviation (RMSD) value of 0.30 Å was carried out using optimized potentials for liquid simulations (OPLS-2005) force field after assigning charge and protonation state. Ligand preparation was done by using the Schrodinger LigPrep utility (Schrodinger, LLC, USA) which generates a low energy 3d structures.

The active site of protein was defined by a bounding box (grid) that was centered on the native ligand in the crystal complex. Extra-precision glide docking (Glide XP) which docks ligands flexibly was used to rank the docking poses and to gauze the binding affinity of these ligands toward the protein.

#### ADME prediction

ADME properties were predicted for synthesized compounds **1a–1g** using computational methods. In this study, we have calculated QpLogP_o_/w, QP LogHERG, QPPCaco, QpLogBB, QPPMDCK, QPlogKp, QpLogHSA, Percentage Human oral absorption and Lipinski’s rule of five using QikProp.

## Results and discussion

### Chemistry

**Fig 2:**
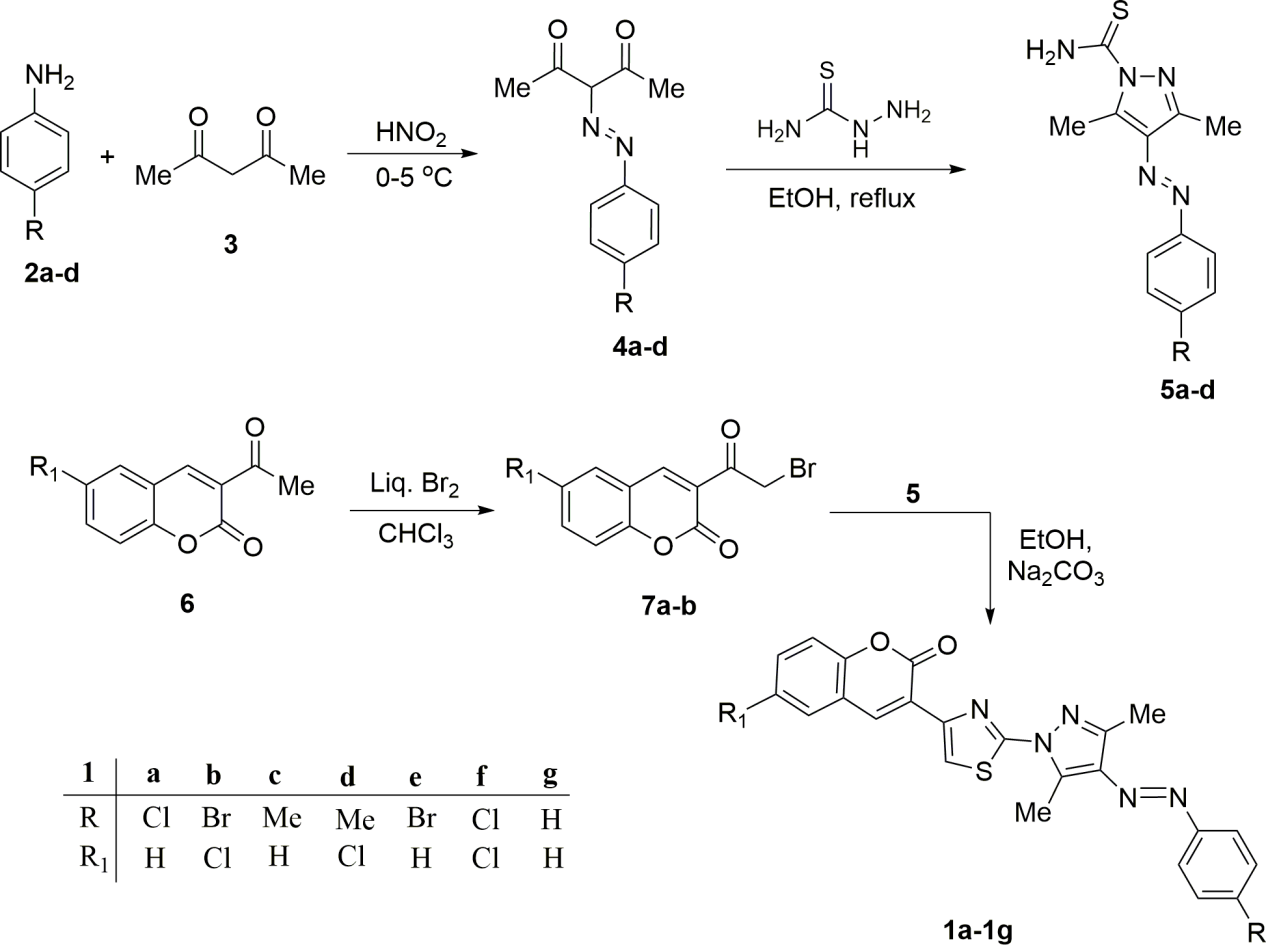
Synthesis of 4-(coumarin-3-yl)-2-(3,5-dimethyl-4-hydrazoarylpyrazol-1-yl)thiazoles (1a-1g).

In view of our aim of building coumarin-thiazole-pyrazole (CTP) based molecular hybrids the coumarin analog i.e., 6-substituted-3-acetylcoumarins (**6**) was synthesised from a reaction of substituted salicylaldehydes with ethyl acetoacetate in the presence of piperidine as catalyst. 6-substituted-3-acetylcoumarins (**6**) thus formed was subjected to bromination using Br_2_/CHCl_3_ to yield 6-substituted-3-bromoacetylcoumarins [52] (**7a-b**) (Fig 2). 3,5-Dimethyl-4-aryldiazenylpyrazole-1-thiocarboxamides (**5a-d**) required for the condensation with 6-substituted-3-bromoacetylcoumarins (**7a-b**) were prepared by the reaction of thiosemicarbazide with **4a-d** according to the previously reported procedures (Fig 2) [53,54]. The compounds **4a-d** were prepared by coupling of diazotized substituted aniline (**2**) with acetyl acetone (**3**) at 0–5°C (Fig 2).

In order to achieve the synthesis of 4-(coumarin-3-yl)-2-(3,5-dimethyl-4-aryldiazenylpyrazol-1-yl)thiazoles(**1a-1g**) exclusively, the condensation of 3,5-dimethyl-4-hydrazoarylpyrazole-1-thiocarboxamides (**5**) with 6-substituted-3-bromoacetylcoumarins (**7a-b**) was carried out in refluxing ethanol under alkaline condition (Fig 2). The structure of all the synthesized compounds were established by analyzing using IR, NMR (^1^H, ^13^C) and mass.

The ^1^H NMR spectra of compounds (**1a-1g**) revealed two singlets in the range δ 8.39–8.29 and 8.00–8.10 ppm corresponds to coumarin 4-H and thiazole 5-H, respectively, thereby confirming the formation of **1**. Two Singlets in the range δ 2.19–2.32 and 2.69–2.86 ppm appeared due to 3-CH_3_ and 5-CH_3_ group attached at pyrazole ring. It has been well established that 5-CH_3_ is more deshielded compared to 3-CH_3_ due to a lone pair of electrons present on the nitrogen atom of the thiazole nucleus and therefore appeared at about δ 2.80ppm [41,55]. Moreover, the appearance of other signals in the NMR spectra corresponding to aromatic protons and carbons furhter establish the structure of the synthesized compounds and complete assignment of ^1^H NMR along with other spectral data has been given in experimental section.

### Biological evaluation

#### Antimicrobial activity

The antimicrobial activity of compounds (**1a-1g**) was evaluated *in vitro* according to Clinical and Laboratory Standard Institute (CLSI) guidelines using five clinical bacterial strains (*Escherichia coli*, *Staphylococcus aureus*, *Enterococcus faecalis*, *Vibrio cholera* and *Streptococcus pyogens*) and six fungal strains (*Candida albicans*, *Candida krusei*, *Candida glabrata*, *Candida keyfer*, *Candida tropicalis*, *Candida parapsilosis*). The antimicrobial activity of synthesized compounds (**1a-1g**) ranged from 250 μM to 15.67 μM and results have been summarized in Tables 1 and 2. The activities and efficacies of the compounds were compared with kanamycin, a routinely used standard antibiotic, and amphotericin B, a well-established antifungal drug [56–58]. Interestingly, the compounds **1b** and **1f** have been found to be the most potent antimicrobial agents among all tested compounds. When used as a standalone antibacterial agent, the MIC of **1b** was either comparable (*S*. *aureus*) or at least 50% reduced (*S*. *pyogens*) compared to KAN. In case of **1f**, the MIC is 50% less compared to that of KAN when tested against *S*. *aureus*, *E*. *faecalis* and *S*. *pyogens*.

**Table 1:** Minimum inhibitory concentration (MIC) of the compounds against five bacterial strains.

Compound	Minimum inhibitory concentration values* (μM)				
	*S*. *aureus*	*E*. *coli*	*E*. *faecalis*	*S*. *pyogens*	*V*. *cholerae*
**1a**	>250	>250	>250	>250	>250
**1b**	**31.25**	>250	250	**31.25**	250
**1c**	>250	>250	>250	>250	>250
**1d**	>250	>250	>250	>250	>250
**1e**	>250	>250	>250	>250	>250
**1f**	**15.67**	62.50	**31.25**	**31.25**	>250
**1g**	>250	>250	>250	>250	>250
KAN^#^	31.25	03.90	62.50	62.50	62.50

*MIC values of the compounds were calculated as CFU plating method as described in materials and methods

^#^KAN, a frequently used antibacterial drug, was used as positive control for this study.

**Table 2:** Minimum inhibitory concentration (MIC) of compounds against six fungal strains.

Compound	Minimum inhibitory concentration values* (μM conc.)					
	*C*. *albicans*	*C*.*glabrata*	*C*.*keyferkeyfer*	*C*. *krusei*	*C*. *tropicalis*	*C*. *parapsilosis*
**1a**	>250	>250	>250	>250	>250	>250
**1b**	**62.50**	250	**62.50**	125	125	62.50
**1c**	>250	>250	>250	>250	>250	>250
**1d**	>250	>250	>250	>250	>250	>250
**1e**	>250	>250	>250	>250	>250	>250
**1f**	**62.50**	250	**62.50**	**62.50**	125	62.50
**1g**	>250	>250	>250	>250	>250	>250
Amp B^#^	0.78	0.78	12.50	0.78	0.78	0.78

^#^Amp B, was used as positive control for this study

*MIC values of the compounds were calculated as CFU plating as described in materials and methods

Though MIC was much higher in case of antifungal screening, the compounds **1b** and **1f** as standalone found to be more active in comparison to other candidates when Amp B was used as reference drug. The results of antimicrobial activity study revealed that incorporation of halogen atoms (Cl and Br) on coumarinyl and phenyl moieties linked to pyrazolylthiazoles may be responsible for higher potential of **1b** and **1f**. Generally, substitution of hydrogen by halogen in coumarin derivatives compounds increases the lipophilicity and thus increases the rate of cell penetration, which is an important feature of antimicrobial drug efficiency [59]. Substitution of chlorine at position-6 of coumarin ring in **1b** and **1f** significantly increased activity against the bacterial and fungal strains. The results demonstrated that the antimicrobial activity of the investigated compounds was influenced by the physicochemical properties of the type and position of substituent on the aromatic ring.

Furthermore, it is noteworthy here that *S*. *aureus* and *candida* sp. are responsible for major opportunistic infections in clinical settings. There are growing concerns about the ability of *S*. *aureus* to acquire resistance, even towards the newest antibacterial available in the market [60]. Similarly, *C*. *keyfer* infections mostly occur in patients with hematologic malignancies or carcinomas who are already undergoing highly cytotoxic chemotherapy [61]. KAN has been used as a standard drug in infections with *S*. *aureus* [62]. Likewise, Amp B is a one of the primary standard drugs used in case of candida infections [61,63]. Emergence of resistance against these front line drugs has been a major public health problem. An important strategy to reduce the emergence of drug resistant strains is by reducing the effective amount of drug required to kill the infectious bacterial or fungal agent [64,65]. Therefore, combinatorial therapies or use of adjuvant is encouraged or used to modulate the antimicrobial activity and mitigate the possibility of development of drug resistance [46].

#### Synergistic effect

As discussed above, we further evaluated whether the compounds **1b** or **1f** can act as an adjunct to potentiate a reduction in MIC of KAN and Amp B against clinical strains *S*. *aureus* and *C*. *Keyfer*, respectively. A checkerboard dilution assay has been used to determine the fraction inhibitory concentration index (FICI) for the compounds **1b** and **1f**, when used in combination with KAN and Amp B against *S*. *aureus* and *C*. *Keyfer*, respectively. As evident from Table 3, we observed an FICI of ≤ 0.5 in all drug combinations reported here indicating a synergistic interaction between the compounds and known drugs. There was a substantial 8-fold reduction in the MIC of KAN against *S*. *aureus* with compounds **1b**, and also with **1f**. Likewise, in case of Amp B, there is a 5-fold reduction in MIC with compound **1b** and ~8 fold reduction with compound **1f** against fungal pathogen *C*. *keyfer*.

**Table 3:** Synergistic effect of compounds 1b and 1f with antibacterial and antifungal drugs against *S*. *aureus and C*.*keyfer*.

Antibacterial/ Antifungal drug (μM)	Compd. (μM)	Conc. (μM) of the drug in combination with 1b or 1f	Fraction inhibitory conc. index (FICI)^a^	Effect
4.0 (KAN)	31.25 (**1b**)	0.5 (KAN)+7.81 (**1b**)	0.500	Synergy
4.0 (KAN)	15.67 (**1f**)	0.5 (KAN)+3.91 (**1f**)	0.374	Synergy
12.50 (Amp B)	62.50 (**1b**)	3.12 (Amp B)+7.81 (**1b**)	0.364	Synergy
0.40 (FLC)	62.50 (**1b**)	0.08 (FLC)+15.62 (**1b**)	0.450	Synergy
12.50 (Amp B)	62.50 (**1f**)	1.56 (Amp B)+6.25 (**1f**)	0.240	Synergy
0.40 (FLC)	62.50 (**1f**)	0.05 (FLC)+15.67 (**1f**)	0.375	Synergy

^a^The fraction inhibitory concentration index (FICI) value is defined as the combination of two active agents can exert inhibitory effects that are more than the sum of their effect alone.

#### Cytotoxic evaluation

**Fig 3:**
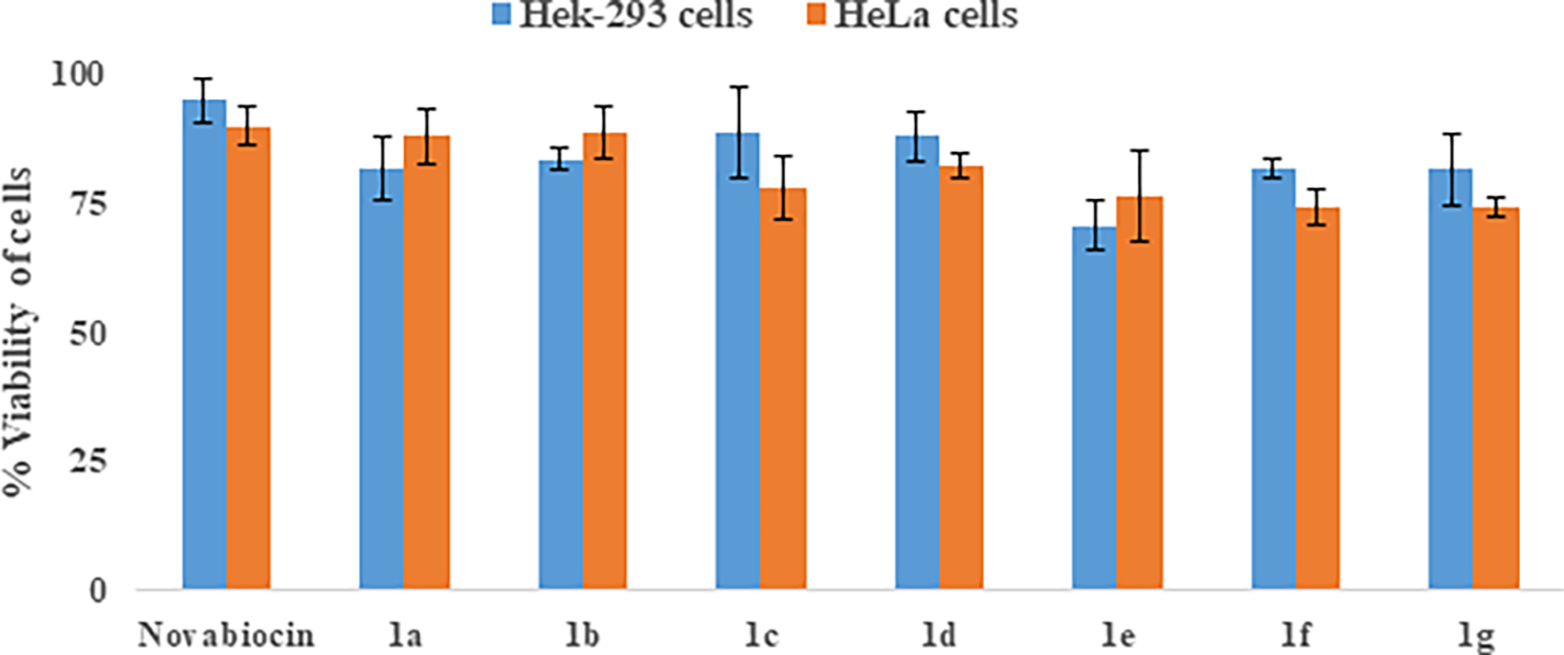
Evaluation of cytotoxicity. Cell toxicity of compounds 1a-1g in term of viability of Hek-293and HeLa cells. Data represent results from three independent experiments. Novobiocin was used as a positive control.

Evaluation of cytotoxicity using HeLa cells (cervical cancer cell) and Hek-293 cells (normal embryonic kidney cells) indicated that the compounds **1b** and **1f** have comparable cytotoxicity (> 80% survival) at MIC concentrations (Fig 3) and negligible cytotoxicity (> 90% survival) at the concentrations used in combination. Their cytotoxicity was comparable with Novobiocin, a coumarin derivative used as a positive control [48]. Most potent compounds emerging out of our screen **1b** and **1f** have comparable cytotoxicity at their respective MIC concentrations.

Since compounds **1b** and **1f** are active as an adjuvant at much lower concentrations (5–8 time less than their MIC values), we believe that these compounds can act as an ideal adjuvant to increase the efficacy of KAN, Amp B or possibly another drugs too. Our experiments with another antifungal agent fluconazole showed that usage of **1b** and **1f** as adjunct reduced the MIC ~5-fold with **1b** and 8-fold with **1f** against *C*. *keyfer* substantiating the broad spectrum effects of these derivatives as adjunct. Compound **1f** consistently demonstrated the most potent activity as a standalone as well as in combination to other drugs as an adjunct. So we evaluated this compound for further analysis including a time kill kinetic assay.

#### Time kill kinetic

**Fig 4:**
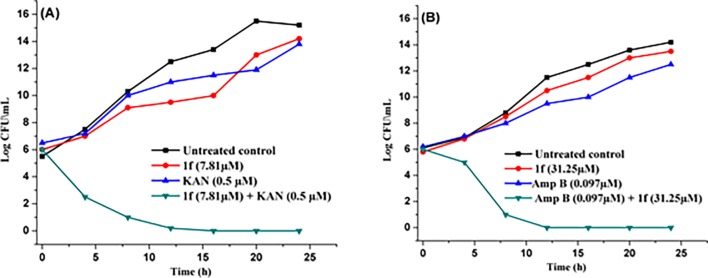
Time kill assay. Impact on growth of (A) *S*. *aureus* by compound **1f** at sub inhibitory conc; (B) *C*. *keyfer* by compound **1f** at sub inhibitory conc.

Time kill kinetic is an important assay to find out the efficacy and killing rate of antimicrobial compounds [66]. In the time kill assay, the Colony Forming Units (CFUs) of the *S*. *aureus* and *C*. *keyfer* were rapidly reduced after treatment with the **1f** (MIC conc. 15.67 μM) and **1f** (MIC conc. 62.50 μM), respectively. Maximum killing of *S*. *aureus* and *C*. *keyfer* cells with **1f** were observed after 24 h in comparison to untreated control. Samples withdrawn at the indicated time points were evaluated for colony forming units (CFUs) at MHA (Muller Hinton Agar) and YPEDA (Yeast Extract Peptone Dextrose Agar) medium. At synergy concentrations of treatment with **1f** (0.5 μM of KAN and 07.81 μM of **1f**) against *S*. *aureus* and (0.097 μM of Amp B and 31.25 μM of **1f**) against *C*. *keyfer* showed killing after 12 h (*S*. *aureus*) and 8 h (*C*. *keyfer*), respectively (Fig 4).

#### *S*. *aureus* and *C*. *keyfer* cells lysis by electron microscopy

**Fig 5:**
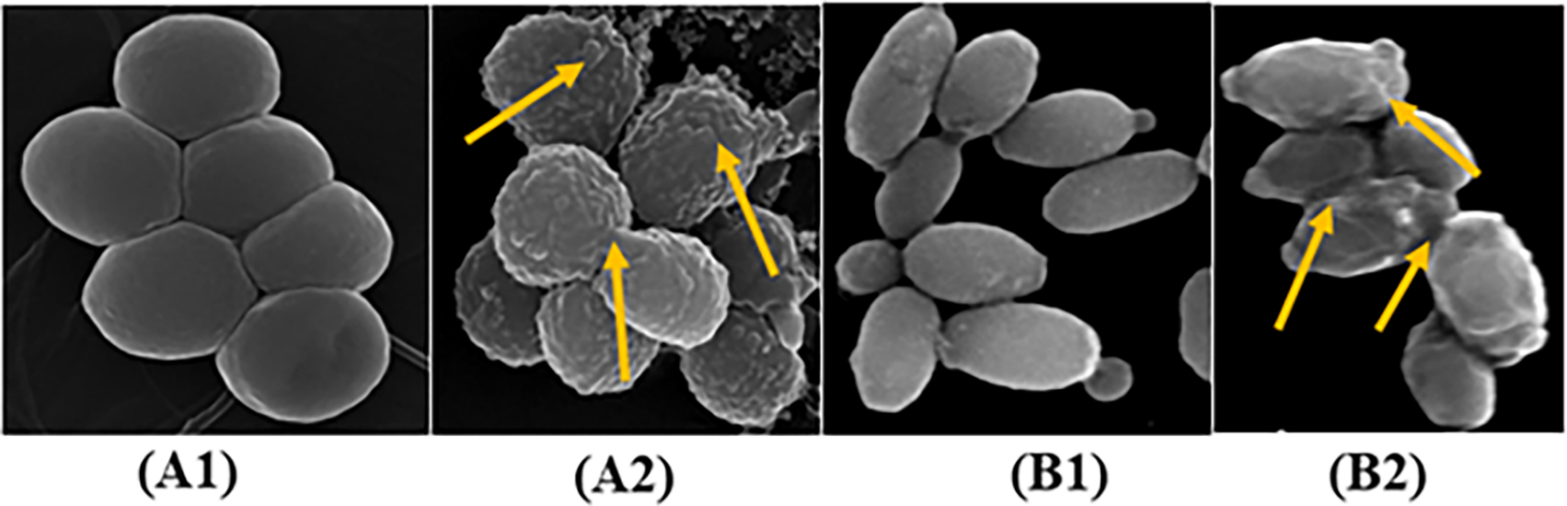
SEM images. (A) *S*. *aureus*, (A1) Untreated cells (40 K, 8.2 mm), (A2) Treated cells with **1f** (40 K, 8.1mm), (B) *C*. *keyfer*, (B1) Untreated cells (5 K, 5 mm), (B2) Treated cells (8.5 K, 2 mm). Arrows showed the damage of *S*. *aureus* and C. *keyfer* cells treated with compound **1f**.

The cell surface morphological effects of the compound **1f** on the *S*. *aureus* and *C*. *keyfer* were also investigated by Scanning Electron Microscopy (SEM). A change in both *S*. *aureus* and *C*. *Keyfer* cell morphology was observed in treated with compound **1f** at MIC conc. (at 15.67 μM for *S*.*aureus* and 62.50 μM for *C*. *keyfer*). Increased roughness and cell wall damage was observed within 12 h of incubation (Fig 5). The untreated *S*. *aureus* and *C*. *keyfer* cells serve as control [67].

As a result, compounds **1b** and **1f** in our screen displayed significant broad spectrum antimicrobial activity against clinical strains of bacteria and fungi without any significant mammalian cell cytotoxicity. Comparing the antibacterial activity of coumarin derivatives, **1b** showed the similar level of activity with MIC 31.25 μM and **1f** showed an excellent level of activity with MIC 15.67 μM when compared with standard drug, Kanamycin. Among the synthesized compounds, the **1f** showed the best activity against *S*. *aureus* (MIC 15.67 μM). The MIC value of Amp B against *C*. *keyfer* is 12.50 μM. More promisingly, when used as an adjunct to standard antimicrobial drugs, compounds **1b** and **1f** significantly enhanced the efficacy of these drugs KAN, Amp B and FLC by significantly reducing the MIC values of these drugs.

### DFT studies: Electrostatic results

The electron donating and receiving ability of a molecule can be determined by considering HOMO and LUMO energy of that molecule. Higher the energy of HOMO higher will be its electron donating ability. A full geometry optimization was performed for all compounds **(1a-1g),** using Khon-Sham’s DFT method that undergo the gradient-corrected hybrid density functional B3LYP method and the 6–31+g(d,p) bases set [49]. It has been found that the energy of HOMO was comparable for all compounds. However, the LUMO orbital energies were affected by the presence of electron donating or withdrawing or both groups on the structure. According to Mabkhot et al. [68], HOMO-LUMO energy gap (E gap) is an established parameter to measure the extent of the intramolecular charge transfer and was used in pharmaceutical studies. In this study, the compounds exhibited different antimicrobial activity, this may be due to the difference in LUMO energy levels. Table 4 summarized the theoretical electronic parameters for investigating the coumarin-thiazole-pyrazole (CTP) based molecular hybrids.

**Table 4:** Theoretical results of compounds 1a-1g.

Compound	Dipole moment (D)	HOMO-1 (eV)	HOMO (eV)	LUMO (eV)	LUMO+1 (eV)	E_gap_ (eV)
**1a**	6.558	-6.528	-6.114	-2.464	-2.435	-3.65
**1b**	4.376	-6.585	-6.175	-2.647	-2.531	-3.528
**1c**	4.108	-6.299	-5.933	-2.366	-2.213	-3.567
**1d**	1.990	-6.530	-5.997	-2.581	-2.268	-3.416
**1e**	6.554	-6.533	-6.107	-2.478	-2.435	-3.629
**1f**	4.390	-6.581	-6.184	-2.648	-2.517	-3.536
**1g**	4.730	-6.368	-6.046	-2.387	-2.301	-3.659

#### Correlation of biological assay with electrostatic results

**Fig 6:**
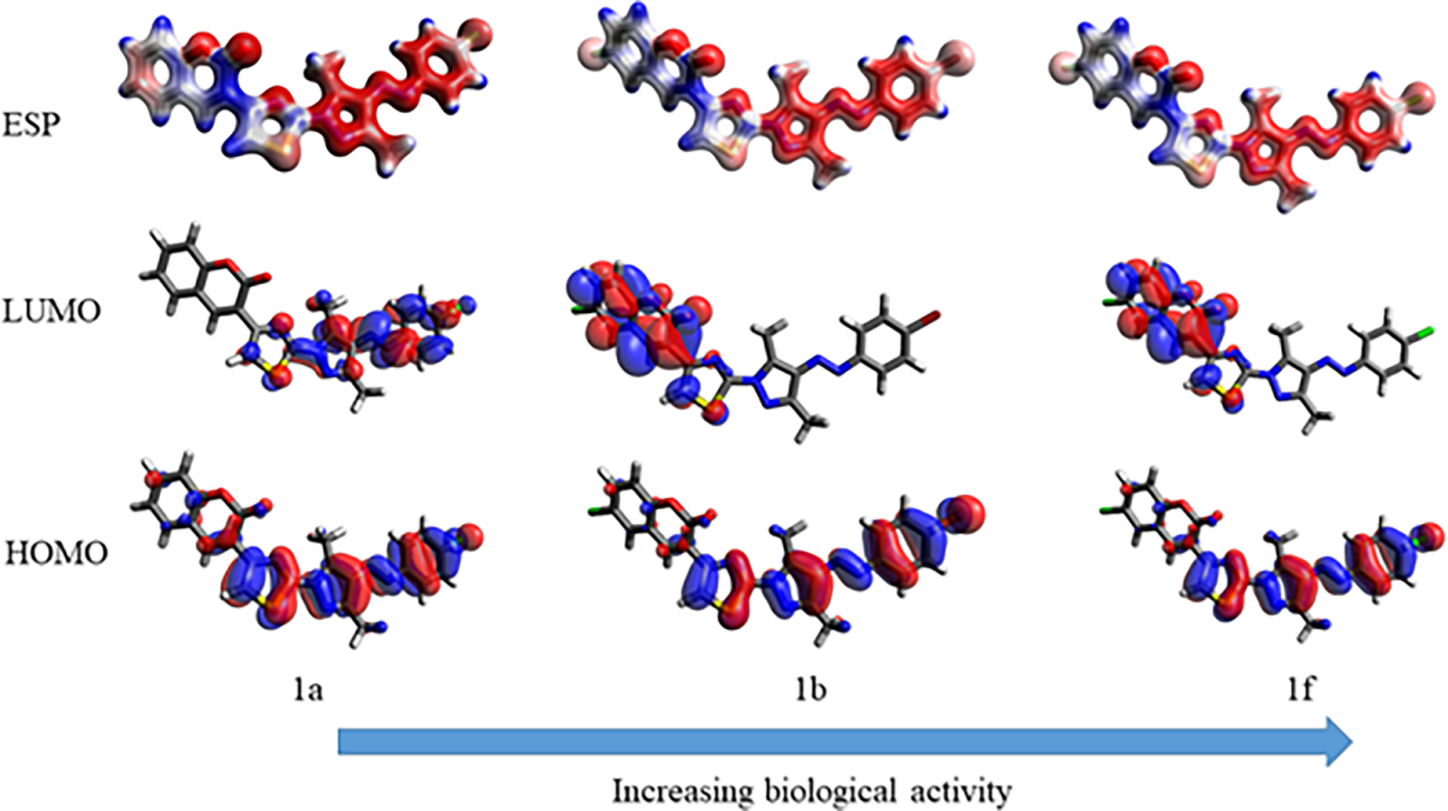
Correlation of biological activity with LUMO energy.

The electrostatic results presented in Table 4 were plotted against the experimental biological activity of synthesized compounds (Fig 6). The biological activity for excellent to low biological active compounds has been correlated with energy of their LUMO. The compound **1b** and **1f** showed illustrious LUMO energy of -2.647 eV and -2.648 eV respectively and also exhibited higher antibacterial & antifungal activity than the other compounds. It was observed that compounds with more stabilized LUMO orbitals have magnificent biological activity (Fig 6).

The effect of LUMO energy on biological activity may be due to the presence of electron withdrawing bromo and chloro groups in compound **1b** and **1f.** Presence of chloro group stabilized LUMO to a higher extent than bromo which was evidenced by the comparison of MIC of compounds **1b** and **1f**. Halogen substitution on coumarin ring stabilized LUMO energy more than on phenyl ring. Relating LUMO energy with biological activity was not the only factor responsible for biological activity. There was a strong demand to look into other aspects like the placement of these molecular orbitals on the molecule (Fig 6). Such investigations revealed that location of LUMO along with their energy together influenced the biological activity. The location of LUMO was particularly affected by the substitution of halogen on coumarin ring which was clearly observed in the correlation of LUMO of compounds **1a**, **1b** and **1f** (Fig 6). It has been revealed from electrostatic results that biological activity of coumarin-thiazole-pyrazole (CTP) hybrids was due to substitution of coumarinyl ring with electron withdrawing groups which consequently lowered its LUMO energy. Therefore, a theoretically suggestion about different biological activity of coumarin-thiazole-pyrazole (CTP) hybrids was made based on the localization and energy of LUMO orbital.

#### Global reactivity descriptors

The chemical reactivity indices like chemical hardness (η), electronegativity (χ), electronic chemical potential (μ), and electrophilicity Index (ω), were also calculated for all the synthesized molecules. The stability and reactivity of a chemical system is represented by chemical hardness which is given by η = (E_LUMO_- E_HOMO_)/2. This descriptor is used as a measures of resistance to change in the electron distribution or charge in a molecule [69]. Electronegativity is given by expression χ = -(E_HOMO_+ E_LUMO_) /2, which is defined as the power of an atom in a molecule to attract electrons towards it [70]. The negative term of electronegativity of a molecule is defined as Chemical potential [71] which is determined using equation μ = (E_HOMO_+E_LUMO_)/2. Electrophilicity index (ω), is a measures of the propensity or capacity of a species to accept electrons was introduced by Parr and is calculated using the electronic chemical potential and chemical hardness and given as ω = μ^2^/2η[72]. The calculated values of chemical reactivity indices for compounds **1a-1f** are summarized in Table 5. The low chemical hardness (1.76 eV) with high negative value of chemical potential (-4.41 eV) indicates that compound **1a** and **1f** are comparatively soft molecule with high polarizability than other compounds of this series. The high electronegativity (4.41 eV) and electrophilicity (5.51 eV) of **1a** and **1f** indicates its high power to withdrawn electrons and hence to act as an electrophile.

**Table 5:** Global reactivity descriptors for compounds *1a-1g*.

Compound	E_H_ (eV)	E_L_(eV)	μ (eV)	χ (eV)	η (eV)	S (eV^-1^)	ω (eV)
**1a**	-6.114	-2.464	-4.289	4.289	1.85	0.2739	5.03
**1b**	-6.175	-2.647	-4.411	4.411	1.76	0.2834	5.51
**1c**	-5.933	-2.366	-4.149	4.149	1.78	0.2803	4.82
**1d**	-5.997	-2.581	-4.289	4.289	1.70	0.2927	5.38
**1e**	-6.107	-2.478	-4.292	4.292	1.81	0.2755	5.07
**1f**	-6.184	-2.648	-4.416	4.416	1.76	0.2828	5.51
**1g**	-6.046	-2.387	-4.216	4.216	1.82	0.2732	4.85

### Docking studies

**Fig 7:**
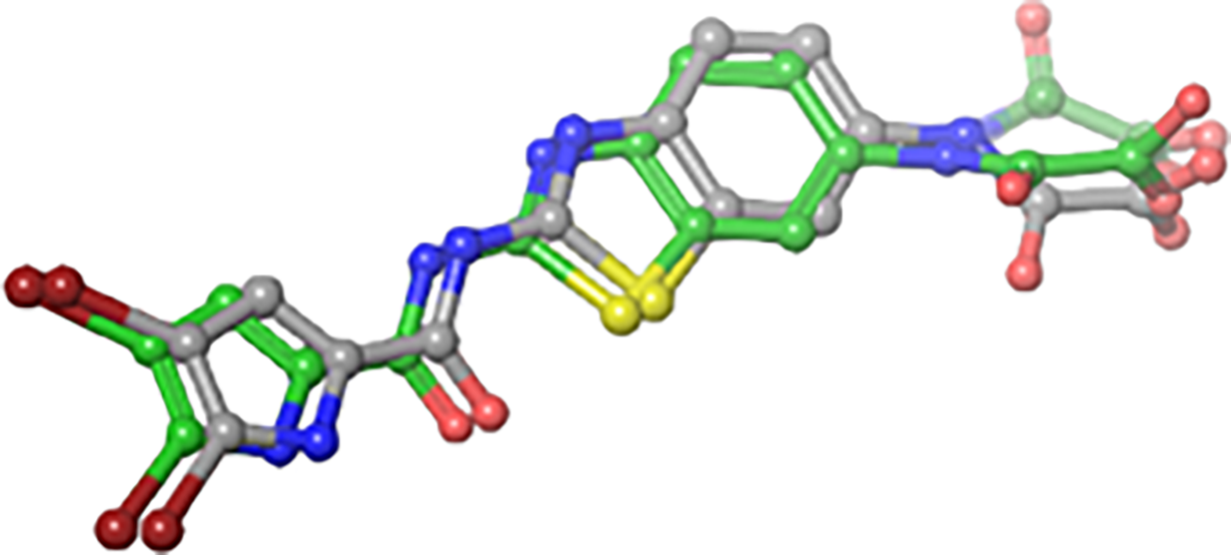
Native ligand (grey) overlap with its best docked pose (green) in a re-docking protocol.

Molecular docking is the most useful technique to explore the possible binding mode between ligand and protein complex. The re-docking of native ligand into the crystal complex is a measure of accuracy of a molecular docking. The comparison between lowest energy pose and the experimentally determined binding mode can be used to as a tool for determining the precision of binding mode. First of all the validation of the docking protocol was done by re-docking the native ligand into the active site of DNA gyrase B (PDB ID: 5L3J) with a root-mean-square deviation (RMSD) of <1Å. This lower value of RMSD represents the accuracy and reliability of the docking procedure in reproducing the experimentally observed results (Fig 7).

**Fig 8:**
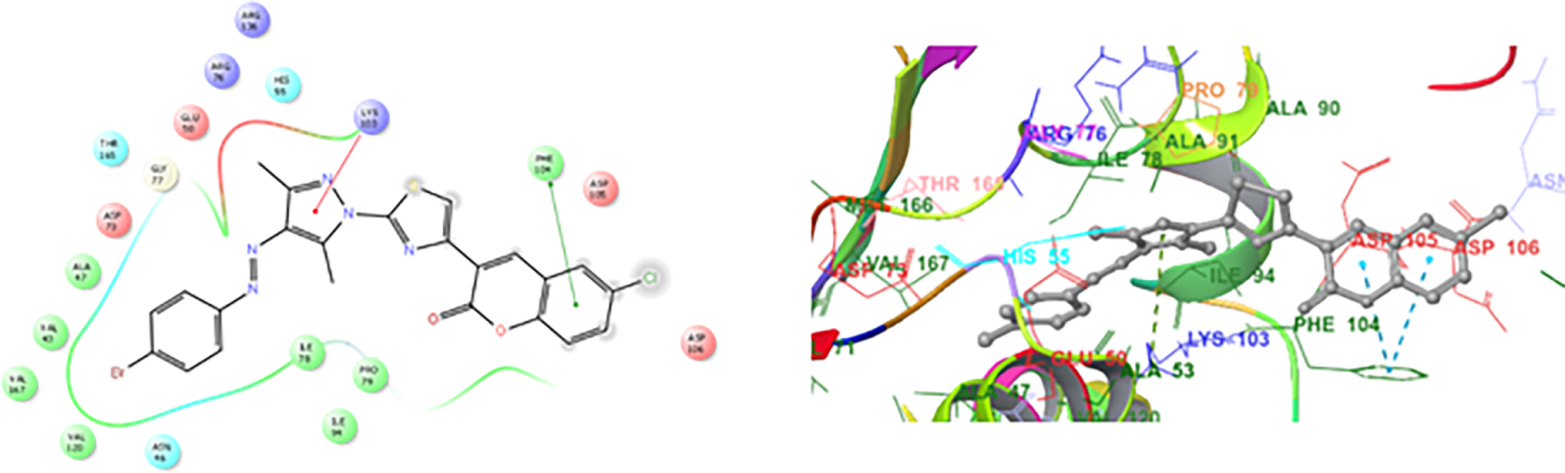
2D and 3D docking pose showing interaction for compound 1b in the binding site of DNA gyrase B crystal structure (PDB ID:5L3J).

**Fig 9:**
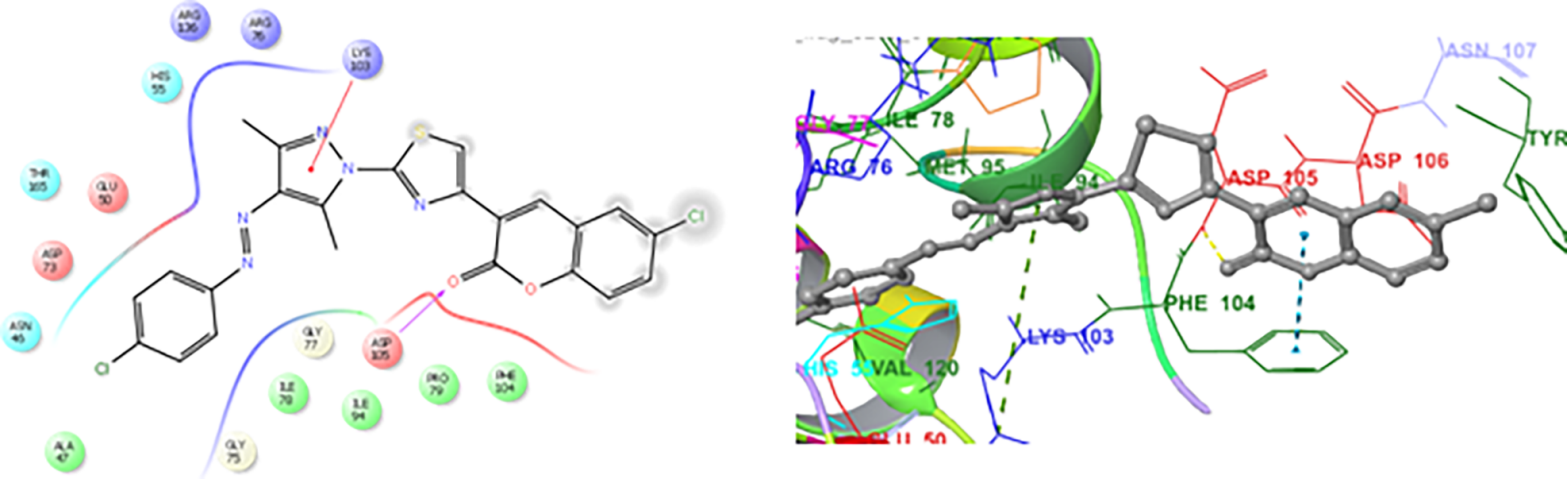
2D and 3D docking pose showing interaction for compound 1f in the binding site of DNA gyrase B crystal structure (PDB ID:5L3J).

**Fig 10:**
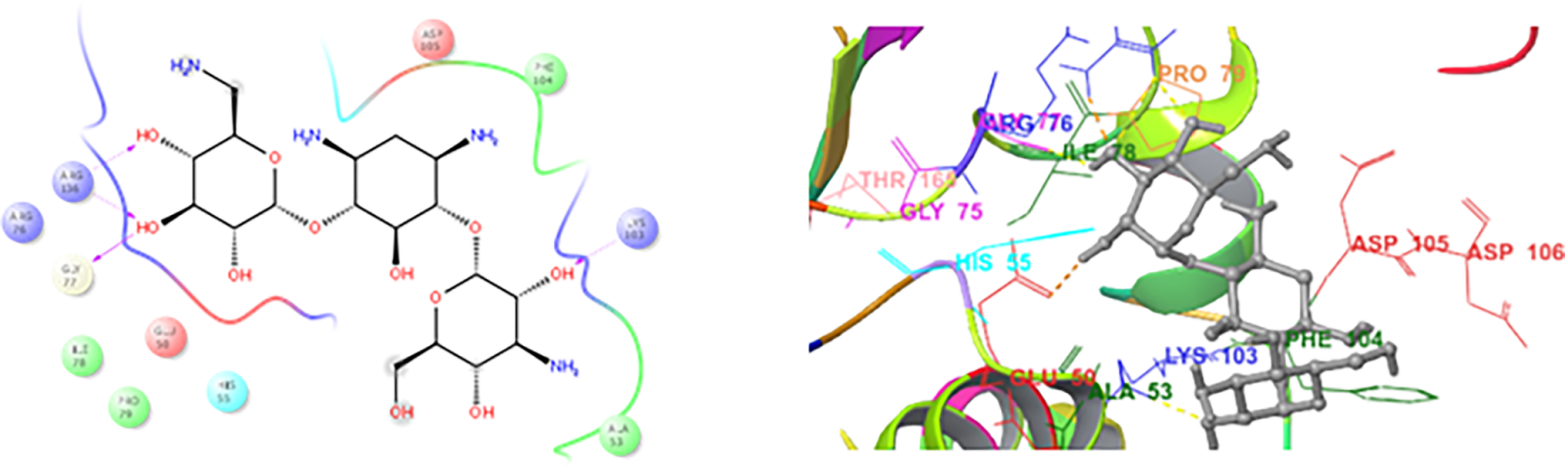
2D and 3D docking pose showing interaction for standard drug KAN in the binding site of DNA gyrase B crystal structure (PDB ID:5L3J).

In the present manuscript, an attempt has been made to study the interactions of novel coumarin-thiazole-pyrazole (CTP) hybrids with DNA gyrase B. For this purpose molecular docking studies of all the synthesized compounds **1a-1g** were performed in the binding pocket of DNA gyrase B (PDB ID:5L3J). Four basic parameters-Glide score, Glide energy, H-bonds and non-bonded interactions (van der Waals and Coulombic) were used to define the binding affinity of ligands **1a-1g** for DNA gyrase B. Glide score with more negative value represents minimum energy for the formation of complex between ligand and receptor (Glide energy). The docked poses for compounds **1b, 1g** and KAN in the active site of DNA gyrase B are shown in Figs 8–10 (Table 6). The most active compounds showed substantial binding affinities towards the DNA gyrase B structures (Glide energy range -69.60 kcalmol^-1^ to -84.78 kcalmol^-1^) and the energy ranges are more than that of reference compounds KAN and CPF (Table 6). It has been revealed from binding studies that all the synthesized compounds **1a-1g** fit snugly making various close contacts with the residues lining the active site of DNA gyrase. In the binding of molecules **1a-1g** to DNA gyrase B, the contribution made by van der waals and columbic interactions are more prevalent over the electrostatic interactions. Extensive van der Waals and columbic interactions have been observed with residues Asn 46, Ile 78, Met 95, Val 120, Val 43, Val 167, Val 71, Thr 165, Ala 47, Glu 50, Gly 75, Asp 73, Gly 77, Arg 76, Pro 79, Asp 105, Ala 90 and Ile 94 lining the active site of DNA gyrase B. Despite these π–π and π-cationic interaction are also present in case of all compounds with Phe 104 and Lys 103 residue respectively. Docked conformations of all compounds (**1a-1g**) with DNA gyrase B are shown in S1 Fig and S2 Fig. The binding pattern observed for these coumarin-thiazole-pyrazole (CTP) based hybrids toward active site of DNA gyrase B opens up a new platform structure-based design efforts.

**Table 6:** Binding data of lead compounds based on MMGB-SA binding energy study.

Entry	GScore	δG_Bind (Kcal/mol)	δG_Bind coulomb (Kcal/mol)	δG Bind Hbond (Kcal/mol)	δG_Bind Lipo(Kcal/mol)	δG_Bind vdW (Kcal/mol)	Key Protein ligands interaction
**1a**	-5.58	-84.78	-5.23	-0.33	-45.12	-58.59	Phe 104, Asn 46, Ile 78, Met 95, Val 120, Val 43, Val 167, Val 71, Thr 165, Ala 47, Glu 50, Gly 75, Asp 73, Gly 77, Arg 76, Lys 103, Pro 79, Asp 105, Ala 90, Ile 94.
**1b**	-4.71	-81.17	-4.92	-0.17	-46.15	-58.55	Asp 105, Pro 79, Ile 94, Ile 78, Asn 46, Val 120, Val 167, Val 43, ALA 47, Asp 73, Thr 165, Gly 77, Glu 50, Arg 76, Arg 136, His 55, Lys 103.
**1c**	-5.61	-83.32	-4.85	-0.33	-44.42	-58.49	Phe 104, Asn 46, Ile 78, Met 95, Val 120, Val 43, Val 167, Val 71, Thr 165, Ala 47, Glu 50, Gly 75, Asp 73, Gly 77, Arg 76, Lys 103, Pro 79, Asp 105, Ala 90, Ile 94.
**1d**	-4.81	-69.60	-5.55	-0.19	-37.32	-56.07	Phe 104, Asn 46, Ile 78, Met 95, Val 120, Val 43, Val 167, Val 71, Thr 165, Ala 47, Glu 50, Gly 75, Asp 73, Gly 77, Arg 76, Lys 103, Pro 79, Asp 105, Ala 90, Ile 94, His 55, Arg 136.
**1e**	-5.6	-84.09	-5.33	-0.33	-45.16	-58.76	Phe 104, Asn 46, Ile 78, Met 95, Val 120, Val 43, Val 167, Val 71, Thr 165, Ala 47, Glu 50, Gly 75, Asp 73, Gly 77, Arg 76, Lys 103, Pro 79, Asp 105, Ala 90, Ile 94.
**1f**	-4.74	-73.32	-5.02	-0.19	-40.08	-56.71	Phe 104, Asn 46, Ile 78, Thr 165, Ala 47, Glu 50, Gly 75, Asp 73, Gly 77, Arg 76, Lys 103, Pro 79, Asp 105, Ala 90, Ile 94, His 55, Arg 136
**1g**	-5.69	-83.22	-5.04	-0.33	-43.65	-57.87	Phe 104, Ile 78, Asn 46, Met 95, Val 120, Val 43, Val 167, Val 71, Thr 165, Ala 47, Glu 50, Gly 75, Asp 73, Arg 76, Gly 77, Lys 103, Pro 79, Asp 105, Ala 90, Ile 94.
**KAN**	-4.74	-27.14	-28.36	-3.72	-15.85	-23.81	Asp 105, Phe 104, Lys 103, Ala 53, His 55, Glu 50, Pro 79, Ile 78, Gly 77, Arg 76, Arg 136.
**CPF**	-5.11	-45.97	-1.83	-0.88	-31.81	-31.63	Phe 104, Asn 46, Ile 78, Thr 165, Ala 47, Glu 50, Gly 75, Asp 73, Gly 77, Arg 76, Lys 103, Pro 79, Asp 105, His 55, Arg 136, Ile 94.

#### Pharmacokinetic effect of synthesized compounds 1a-1g

Pharmacodynamics results indicated that compounds **(1a-1g)** may prove good lead as antibacterial drugs by targeting DNA gyrase B. For drug formulation pharmacokinetic properties are likewise a significant prospect and should be brought into thoughtfulness. The pharmacokinetic parameters of all synthesised hybrids have been studied along with standard drug KAN and CPF using QikProp [73]. The major parameters studied for different synthesised compounds **(1a-1g)** were seems to be acceptable and were incorporated in Table 7. The % oral drug absorption predicted for all the test compounds **1a-1g** was highly satisfactory with a high percentage (>80%) of Human Oral Absorption as compared to the reference, indicating their possibilities in oral drug formulation for the treatment of *S*. *aureus* infection.

**Table 7:** Predicted ADME properties of synthesized compounds 1a-1g and standard drug KAN and CPF.

Compounds	QP logPo/w^a^	QP logHERG^b^	QPPCaco^c^	QP logBB^d^	QPPMDCK^e^	QPlogKp^f^	QPlogKhsa^g^	Percent Human Oral Absorption^h^	Lipinski’s rule of five
**Acceptable range**	**(-2.0to6.5)**	**> -5.0**	**<25 poor > 500 great**	**(-3 to 1.2)**	**<25 poor > 500 great**	**(-8.0 to -0.1)**	**(-1.5 to 1.5)**	**> 80% High, > 25% low**	**< 5**
**1a**	4.96	-7.25	1234.4	-0.36	2402.5	-1.59	0.59	100	0
**1b**	5.55	-7.16	1234.5	-0.19	6376.3	-1.76	0.74	88.874	2
**1c**	4.78	-7.24	1234.3	-0.54	973.0	-1.62	0.63	100	0
**1d**	5.28	-7.15	1187.6	-0.41	2307.2	-1.82	0.76	100	1
**1e**	5.03	-7.29	1187.6	-0.37	2481.5	-1.62	0.62	85.565	2
**1f**	5.47	-7.14	1234.5	-0.20	5930.3	-1.76	0.72	100	1
**1g**	4.45	-7.38	1187.6	-0.54	934.7	-1.45	0.46	100	0
**KAN**	-6.58	-6.19	0.031	-2.93	0.009	-11.20	-1.27	0	2
**CPF**	0.27	-3.13	12.6	-0.67	8.25	-6.54	0	48.315	0

^a^Predicted octanol/water partition co-efficient logp.

^b^ Predicted IC_50_ values to block HERG K^+^channels.

^c^ Predicted Caco-2 cell permeability.

^d^ Predicted brain/blood partition coefficient.

^e^ Predicted apparent MDCK cell permeability.

^f^ Predicted skin permeability.

^g^ Predicted binding to human serum albumin.

^h^ Predicted oral absorption of drug in percentage term.

The bowel-blood barrier was predicted using Caco-2 cell permeability (QPPCaco) as model along with Lipinski’s rule of 5 [74] all the test compounds and showed very good values compared to the standard Kanamycin and ciprofloxacin. Further, the prediction for human serum albumin binding using QPlogKhsa, shows that the values for all the inhibitors lie within the expected range (-1.5 to 1.5). Likewise, the brain/blood partition coefficient (QPlogBB) and IC50 value of HERG K+ channel blockage (QPlogHERG) for all compounds shows satisfactory values as compared with reference compounds (Table 7).

## Conclusion

In conclusion, some new coumarinyl linked pyrazolylthiazoles have been synthesized and explored for their potential as standalone or adjuvant antimicrobial agents against pathogenic strains. Compounds **1b** and **1f** displayed significant antimicrobial activity without expressing any significant mammalian cell cytotoxic effect. Compound **1b** showed the similar level of antibacterial activity with MIC 31.25 μM and **1f** found best agent with MIC 15.67 μM particularly against *S*. *aureus* when compared with standard drug, kanamycin. Interestingly, as an adjuvant to standard antimicrobial drugs, compounds **1b** and **1f** significantly enhanced the efficacy of the standard drugs KAN, Amp B and FLC by significantly reducing the MIC values of these drugs.

The results demonstrated that the antimicrobial activity of the investigated compounds was influenced by the physicochemical properties of the type and position of substituent on the aromatic ring. The results based on electron microscopic investigations of cell morphology and cellular structural integrity suggest that the antimicrobial activity of these compounds may in part be attributed to initial cell wall damage, leading to action on the intracellular components of cells, ultimately leading to cell death. The result of DFT studiy of compounds were correlated with the observed antimicrobial activities which indicate that coumarin moiety plays an important role in antimicrobial activities. Moreover, the molecular docking studies further helped in supporting the experimental results and the ADME properties predicted further strengthen the path towards oral drug formulation. Therefore, it is concluded that the compounds **1b** and **1f** may act as lead compounds for the development of potent antibacterial and antifungal agents and as adjuvant they have potential to unlock the microbial resistance to antibiotics.

## Supporting information

S1 Fig 2D docking pose showing interaction for compound 1a-1g in the binding site of DNA gyrase B crystal structure (PDB ID:5L3J). (TIF)

S2 Fig 3D docking pose showing interaction for compound 1a-1g in the binding site of DNA gyrase B crystal structure (PDB ID:5L3J). (TIF)

S3 Fig ^1^H NMR spectrum of 4-(coumarin-3-yl)-2-(3,5-dimethyl-4-(4-chlorophenyl) diazenyl)pyrazol-1-yl)thiazole (1a). (TIF)

S4 Fig ^1^H NMR spectrum of 4-(6-chlorocoumarin-3-yl)-2-(3,5-dimethyl-4-(4-bromophenyl)diazenyl)pyrazol-1-yl)thiazole (1b). (TIF)

S5 Fig ^1^H NMR spectrum of 4-(6-chlorocoumarin-3-yl)-2-(3,5-dimethyl-4-(4-methylphenyl)diazenyl)pyrazol-1-yl)thiazole (1d). (TIF)

S6 Fig ^1^H NMR spectrum of 4-(coumarin-3-yl)-2-(3,5-dimethyl-4-(4-bromophenyl) diazenyl)pyrazol-1-yl)thiazole (1e). (TIF)

S7 Fig ^1^H NMR spectrum of 4-(6-chlorocoumarin-3-yl)-2-(3,5-dimethyl-4-(4-chlorophenyl)diazenyl)pyrazol-1-yl)thiazole (1f). (TIF)

S8 Fig ^13^C NMR spectrum of 4-(Coumarin-3-yl)-2-(3,5-dimethyl-4-(4-chlorophenyl) diazenyl)pyrazol-1-yl)thiazole (1a). (TIF)

S9 Fig ^13^C NMR spectrum of 4-(6-Chlorocoumarin-3-yl)-2-(3,5-dimethyl-4-(4-bromophenyl)diazenyl)pyrazol-1-yl)thiazole (1b). (TIF)

S10 Fig ^13^C NMR spectrum of 4-(Coumarin-3-yl)-2-(3,5-dimethyl-4-(4-methylphenyl) diazenylpyrazol-1-yl)thiazole (1c). (TIF)

S11 Fig ^13^C NMR spectrum of 4-(Coumarin-3-yl)-2-(3,5-dimethyl-4-(4-bromophenyl) diazenyl)pyrazol-1-yl)thiazole (1e). (TIF)

S12 Fig ^13^C NMR spectrum of 4-(6-Chlorocoumarin-3-yl)-2-(3,5-dimethyl-4-(4-chlorophenyl)diazenyl)pyrazol-1-yl)thiazole (1f). (TIF)

S13 Fig ^13^C NMR spectrum of 4-(Coumarin-3-yl)-2-(3,5-dimethyl-4-phenyldiazenyl) pyrazol-1-yl)thiazole (1g). (TIF)

S14 Fig IR spectrum of 4-(coumarin-3-yl)-2-(3,5-dimethyl-4-(4-chlorophenyl) diazenyl)pyrazol-1-yl)thiazole (1a). (TIF)

S15 Fig IR spectrum of 4-(Coumarin-3-yl)-2-(3,5-dimethyl-4-(4-methylphenyl) diazenylpyrazol-1-yl)thiazole (1c). (TIF)

S16 Fig IR spectrum of 4-(coumarin-3-yl)-2-(3,5-dimethyl-4-(4-bromophenyl) diazenyl)pyrazol-1-yl)thiazole (1e). (TIF)

S17 Fig Mass spectrum for 4-(6-chlorocoumarin-3-yl)-2-(3,5-dimethyl-4-(4-bromophenyl)diazenyl) pyrazol-1-yl)thiazole (1b). (TIF)

S18 Fig Mass spectrum for 4-(coumarin-3-yl)-2-(3,5-dimethyl-4-(4-bromophenyl)diazenyl)pyrazol-1-yl)thiazole (1e). (TIF)

S19 Fig Mass spectrum for 4-(6-chlorocoumarin-3-yl)-2-(3,5-dimethyl-4-(4-chlorophenyl)diazenyl)pyrazol-1-yl)thiazole (1f). (TIF)
