# Supplementary figures and images for: Design, synthesis, DFT, docking studies and ADME prediction of some new coumarinyl linked pyrazolylthiazoles: Potential standalone or adjuvant antimicrobial agents
Source: PLoS One. 2018 Apr 19;13(4):e0196016. doi: 10.1371/journal.pone.0196016 (PMC5908142; doi:10.1371/journal.pone.0196016)

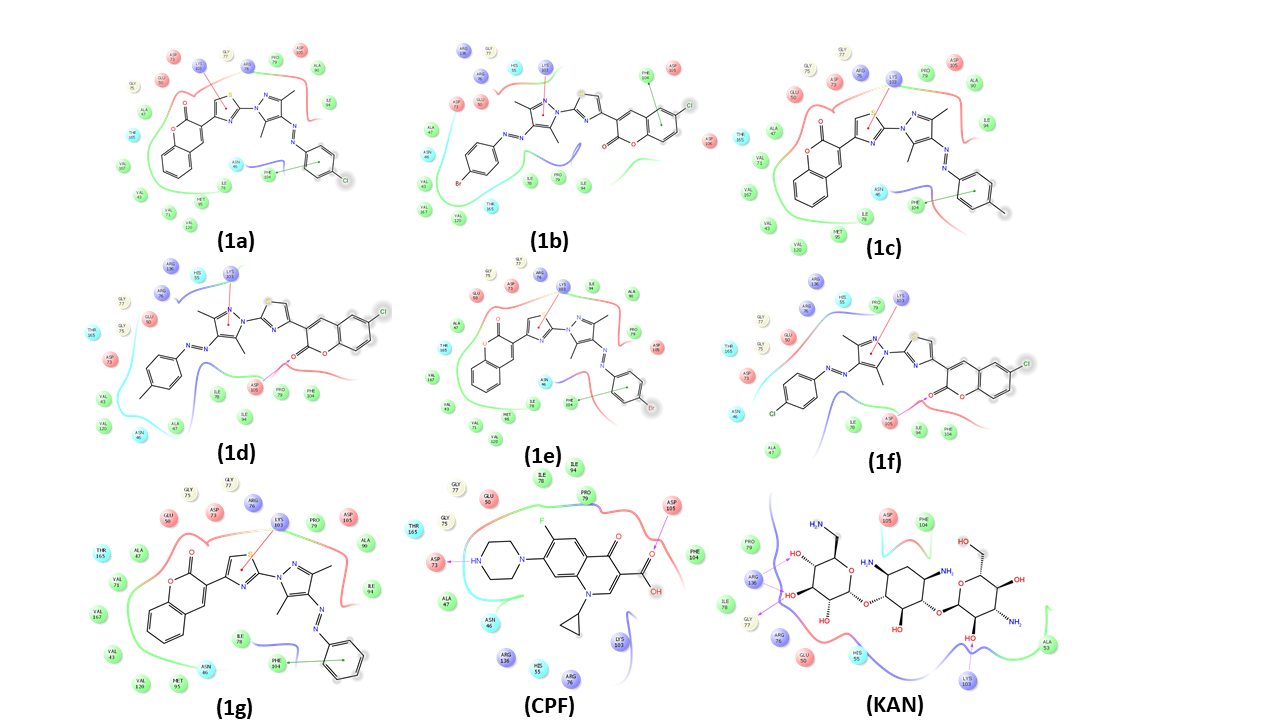

Supplement: S1 Fig — (TIF) [file pone.0196016.s001.tif]

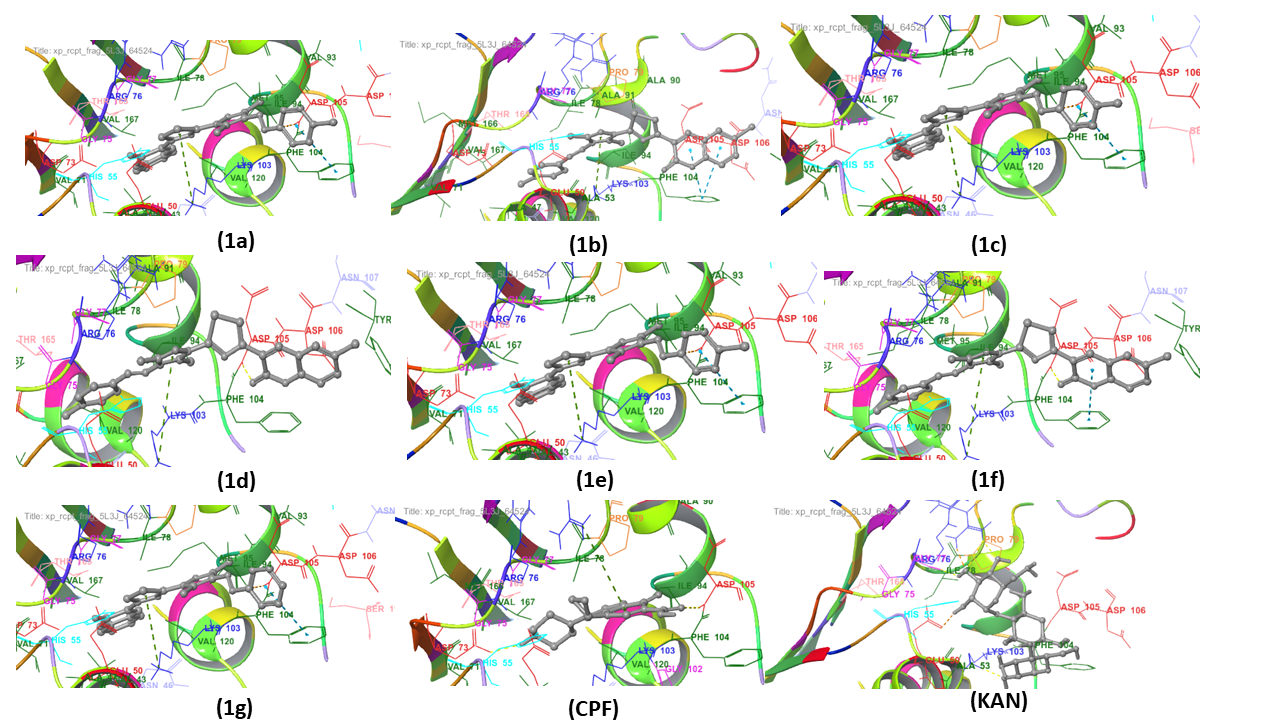

Supplement: S2 Fig — (TIF) [file pone.0196016.s002.tif]

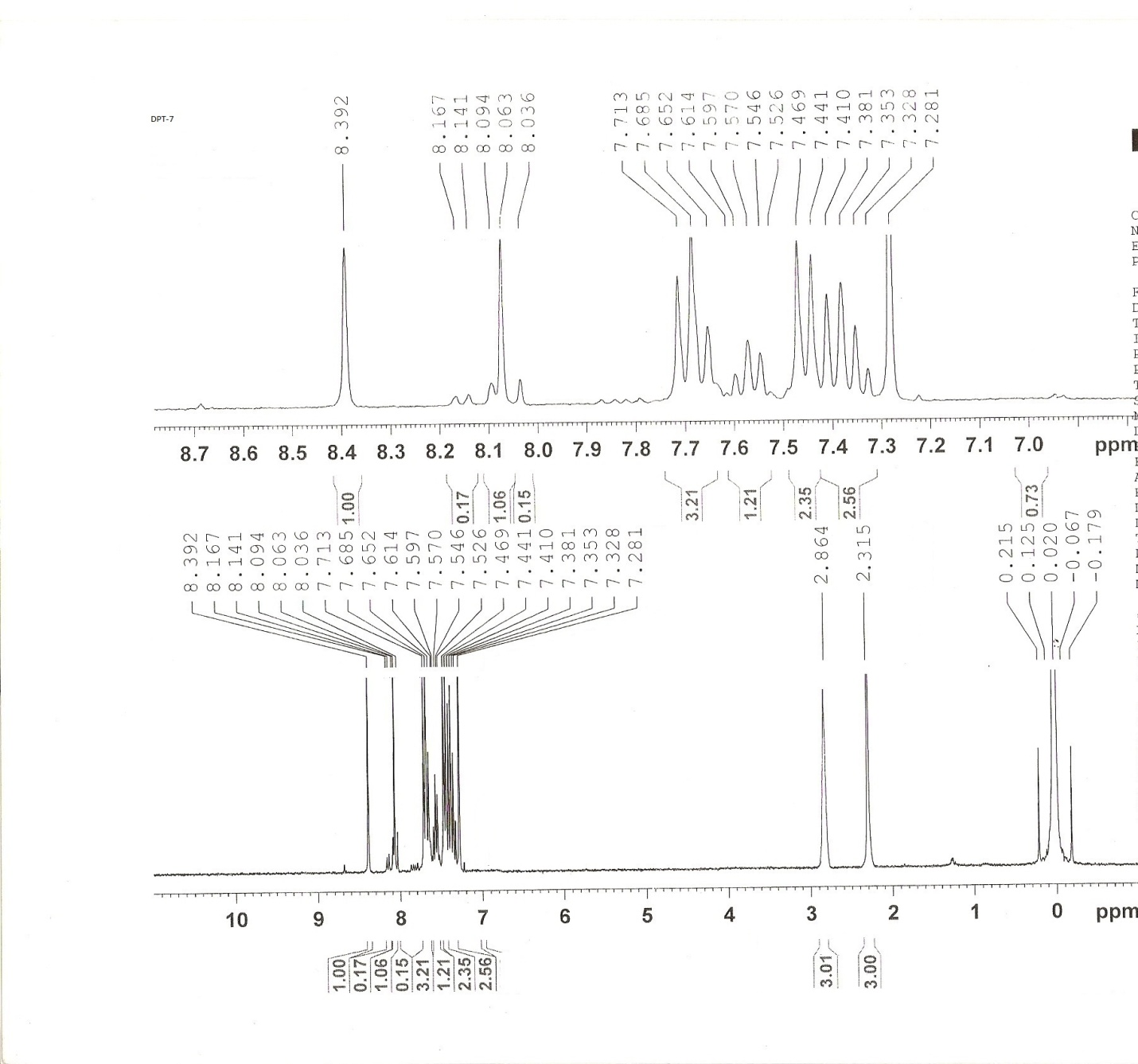

Supplement: S3 Fig — (TIF) [file pone.0196016.s003.tif]

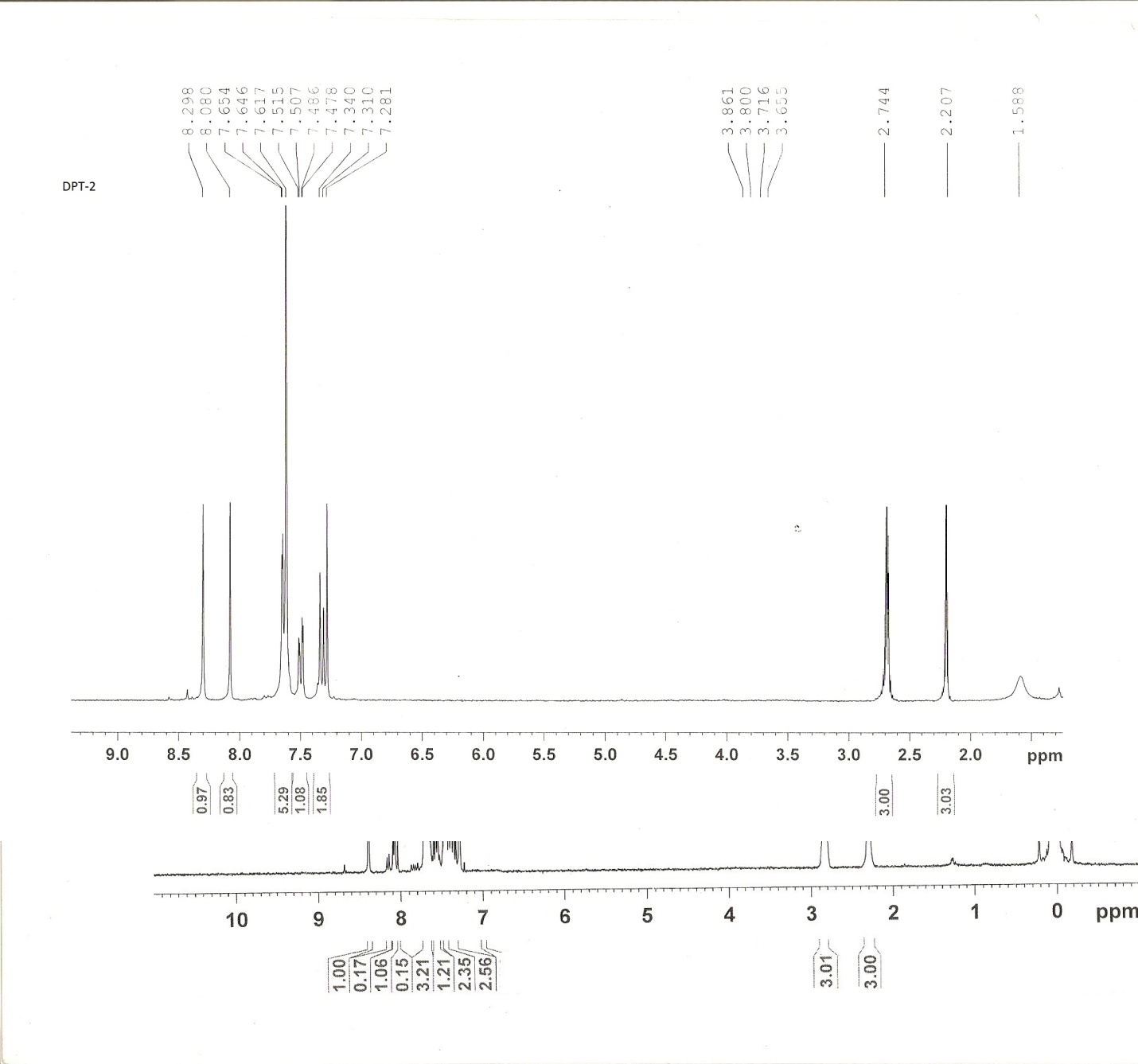

Supplement: S4 Fig — (TIF) [file pone.0196016.s004.tif]

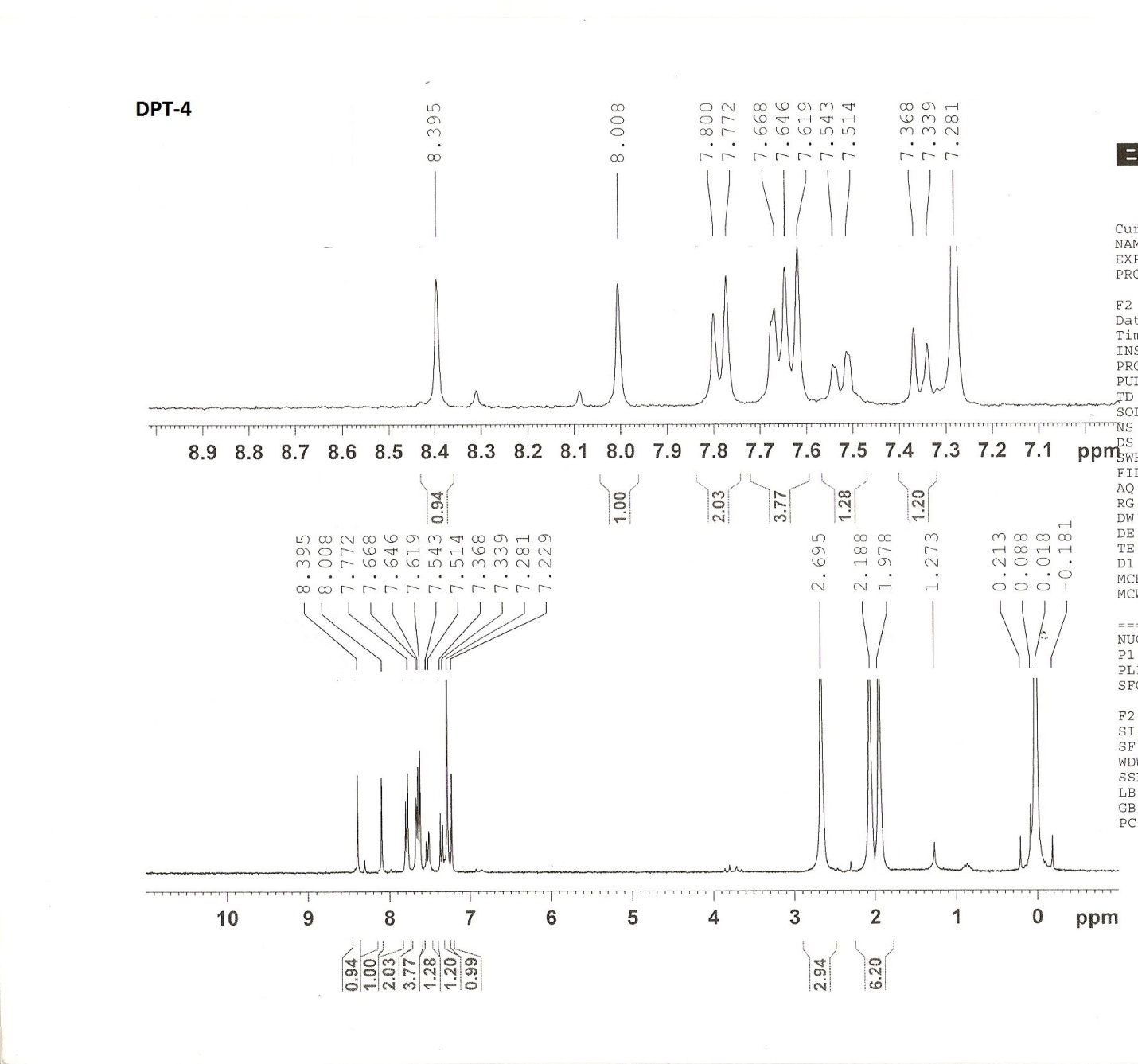

Supplement: S5 Fig — (TIF) [file pone.0196016.s005.tif]

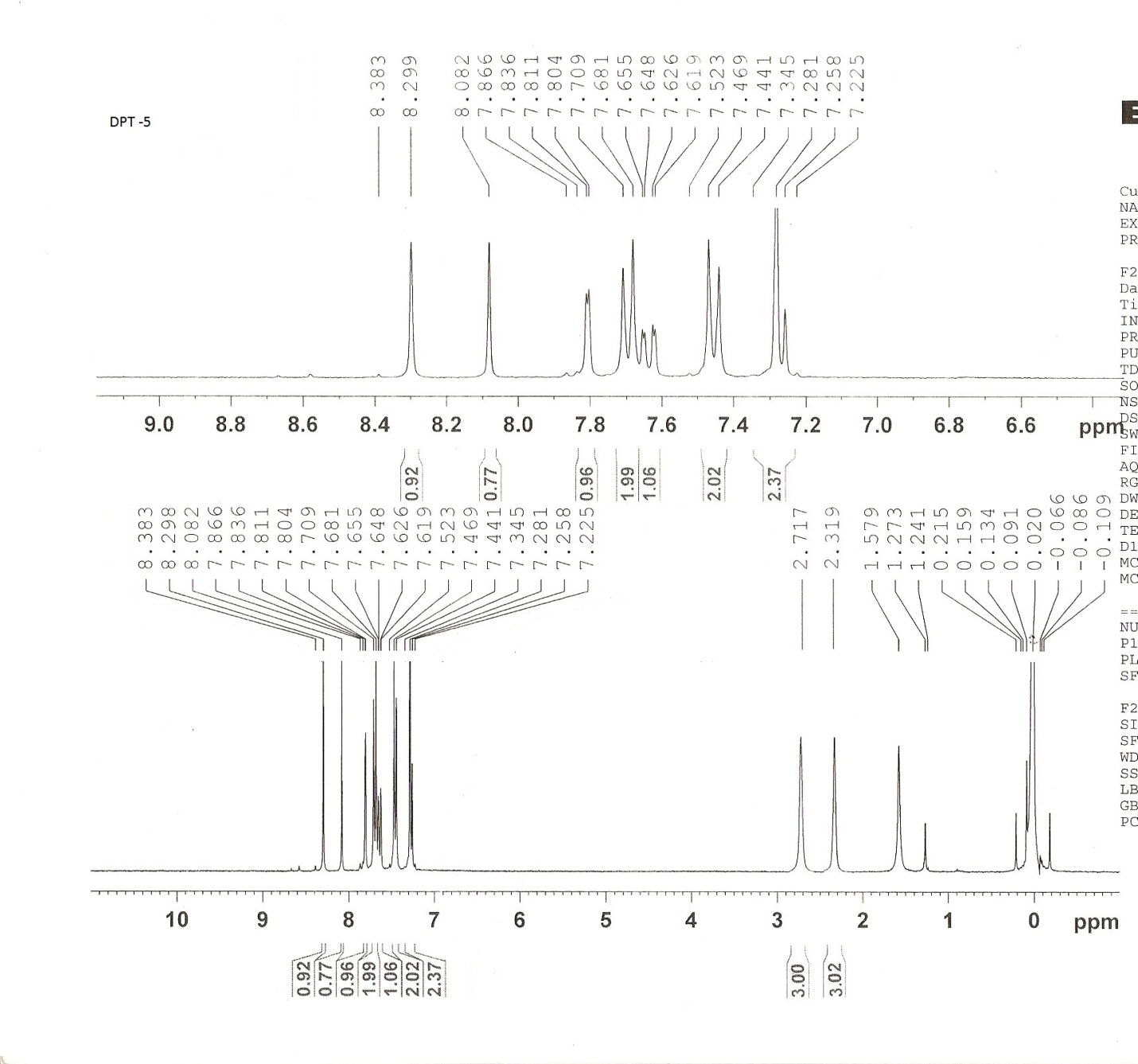

Supplement: S6 Fig — (TIF) [file pone.0196016.s006.tif]

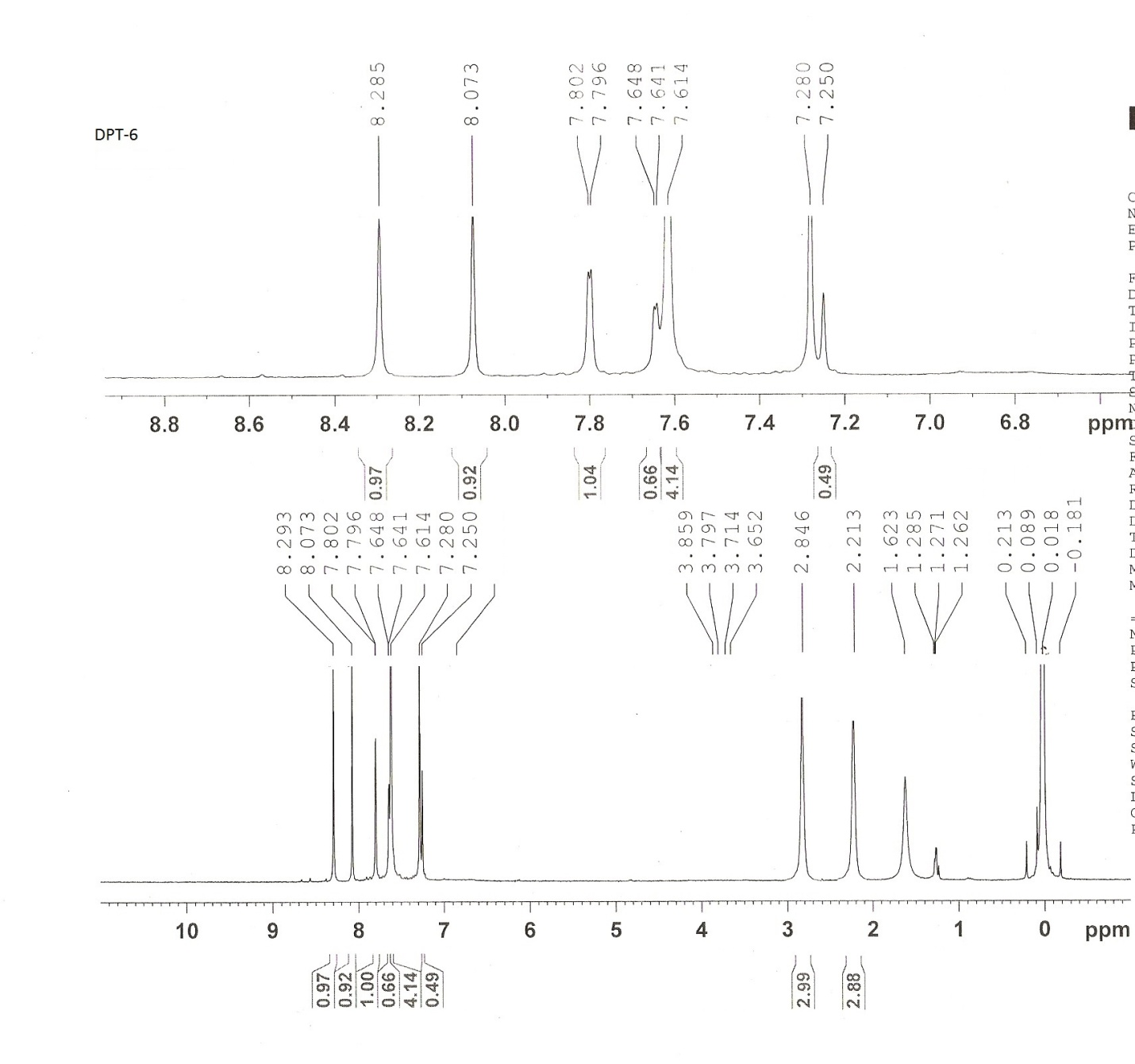

Supplement: S7 Fig — (TIF) [file pone.0196016.s007.tif]

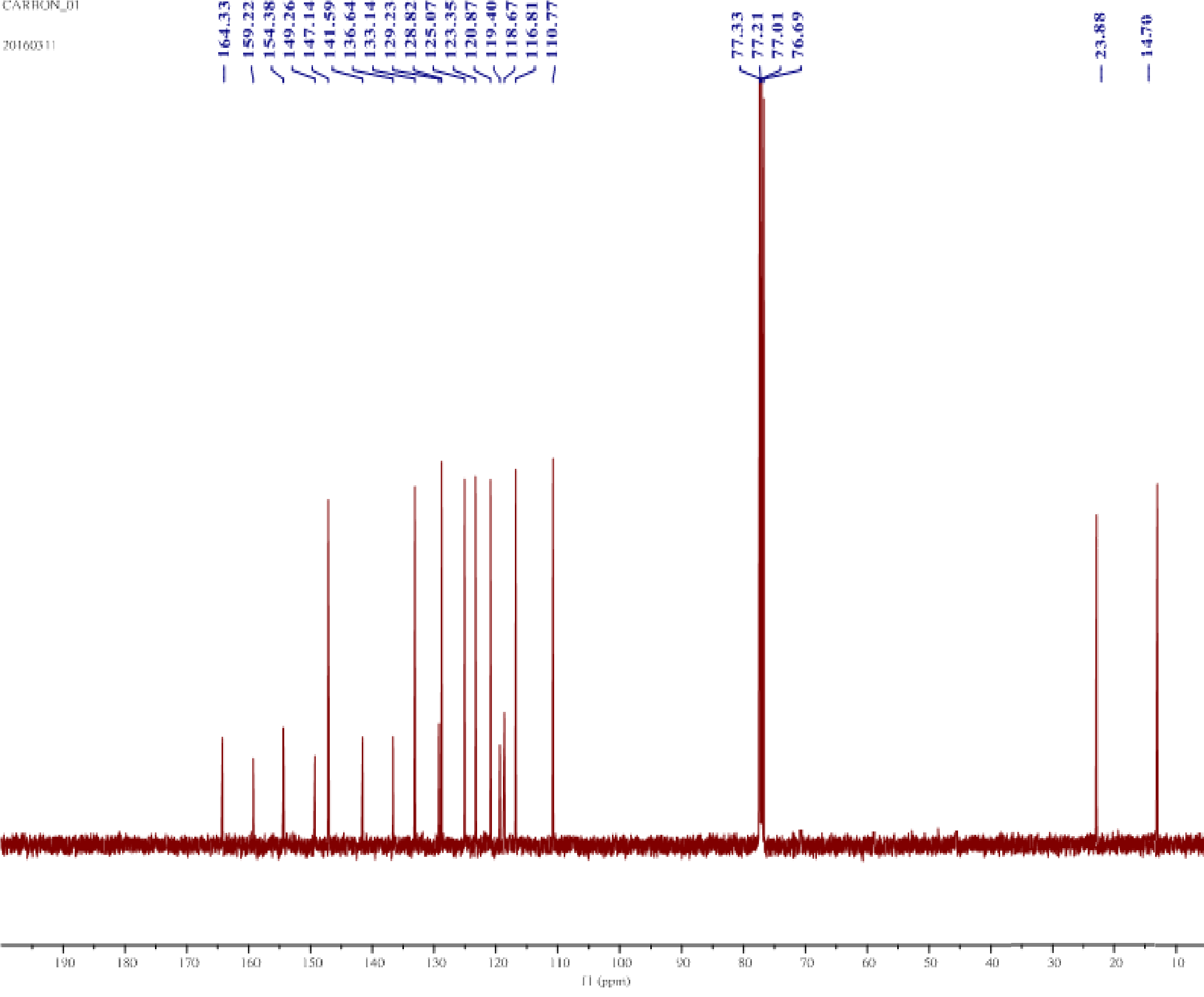

Supplement: S8 Fig — (TIF) [file pone.0196016.s008.tif]

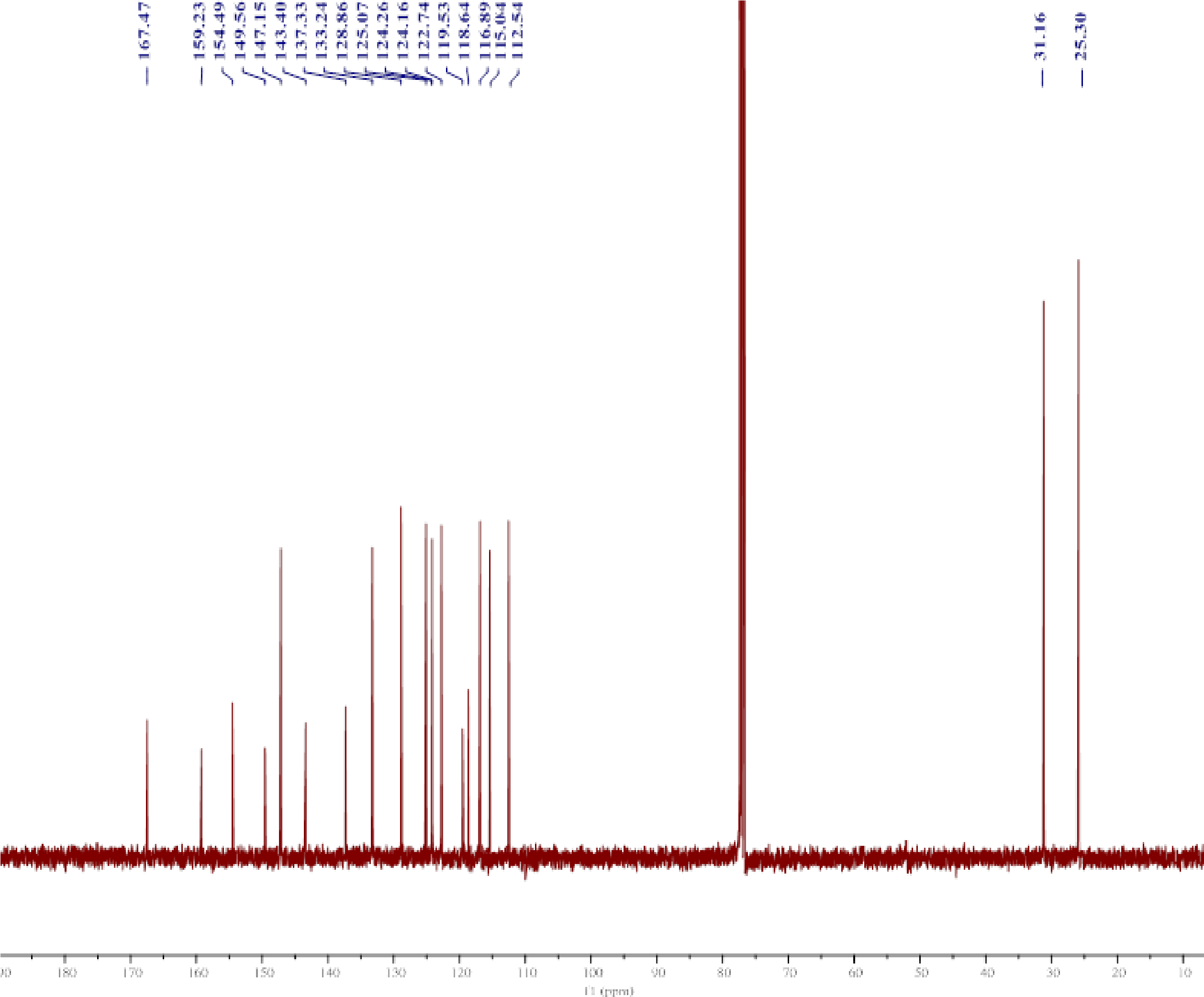

Supplement: S9 Fig — (TIF) [file pone.0196016.s009.tif]

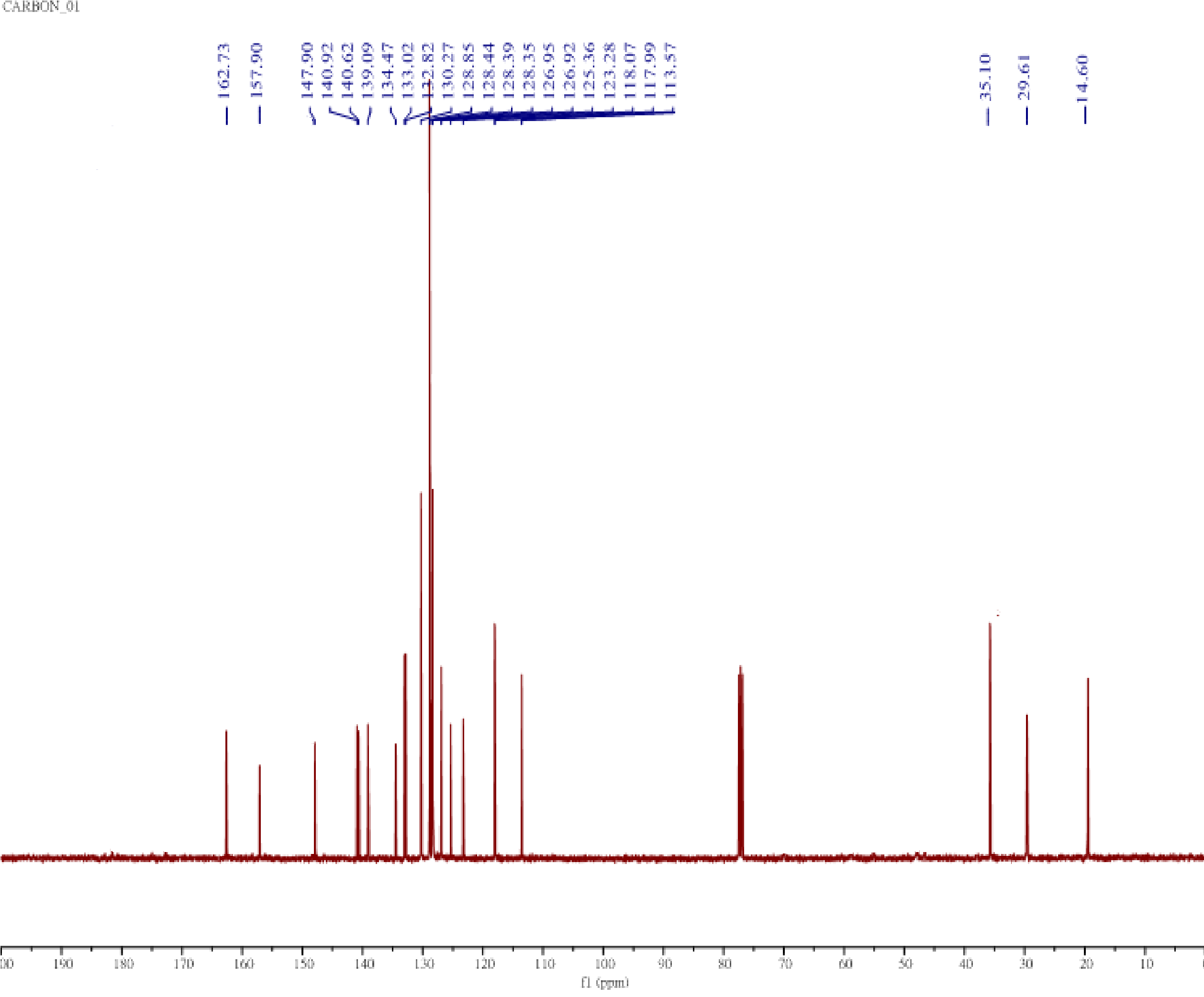

Supplement: S10 Fig — (TIF) [file pone.0196016.s010.tif]

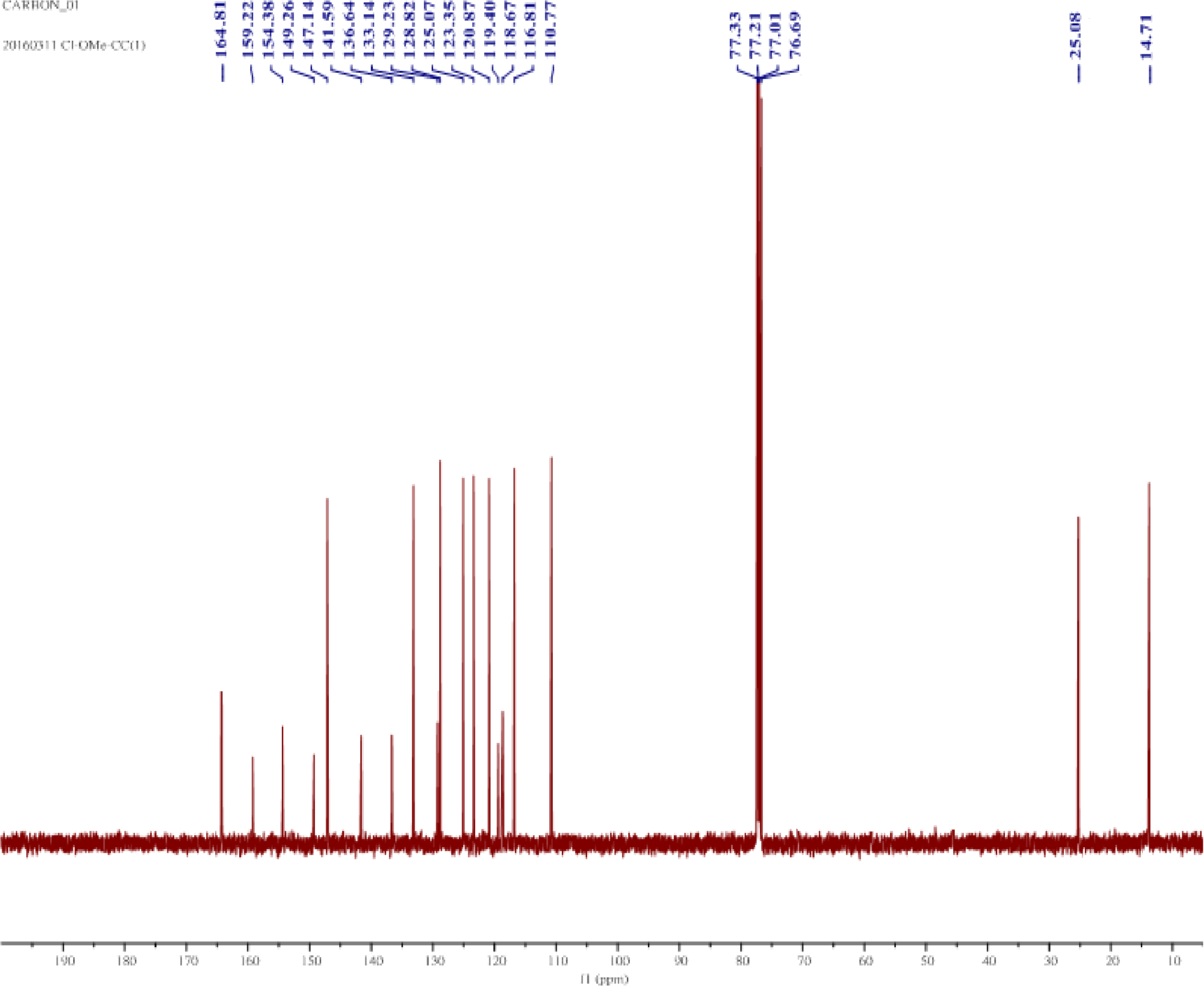

Supplement: S11 Fig — (TIF) [file pone.0196016.s011.tif]

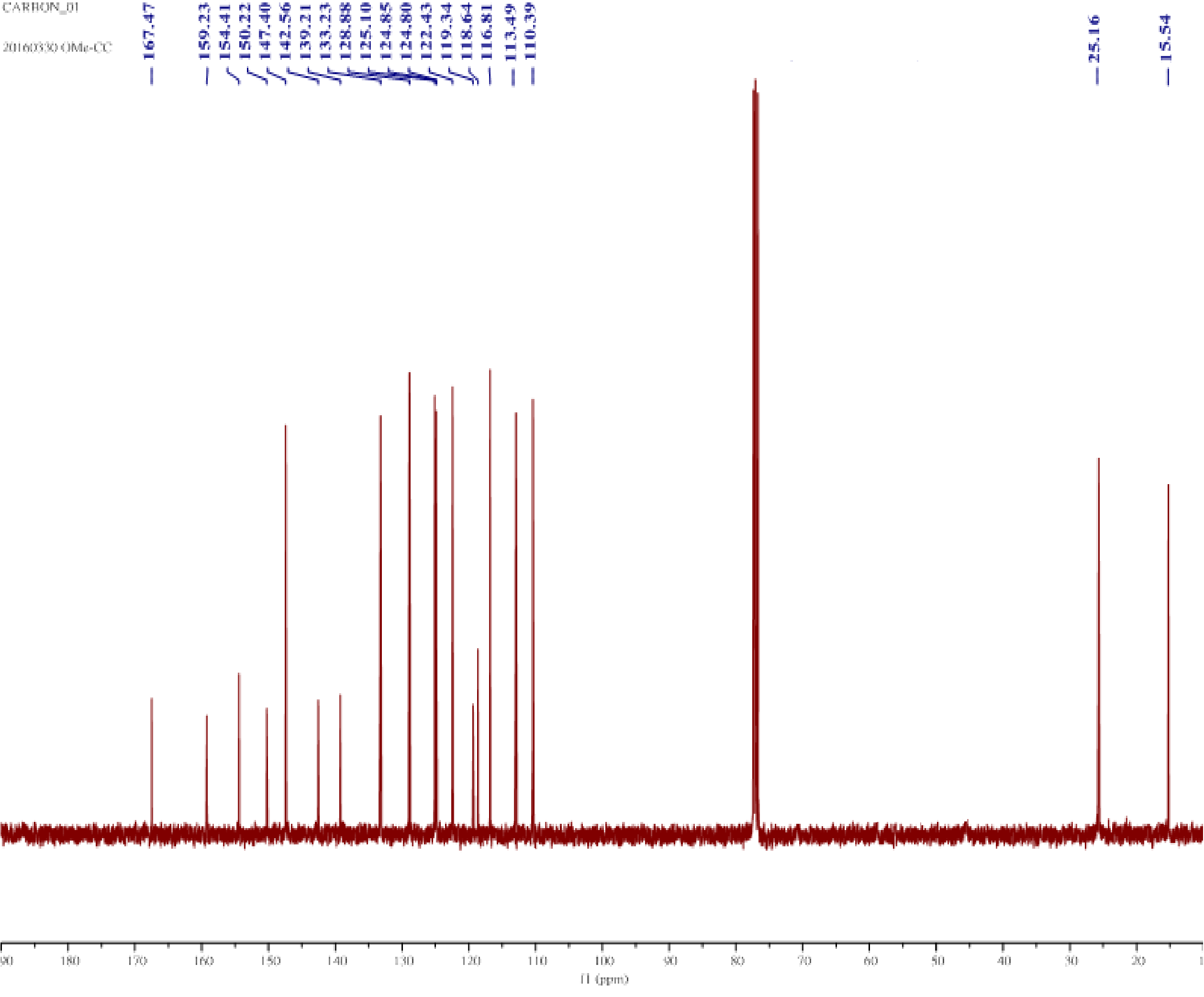

Supplement: S12 Fig — (TIF) [file pone.0196016.s012.tif]

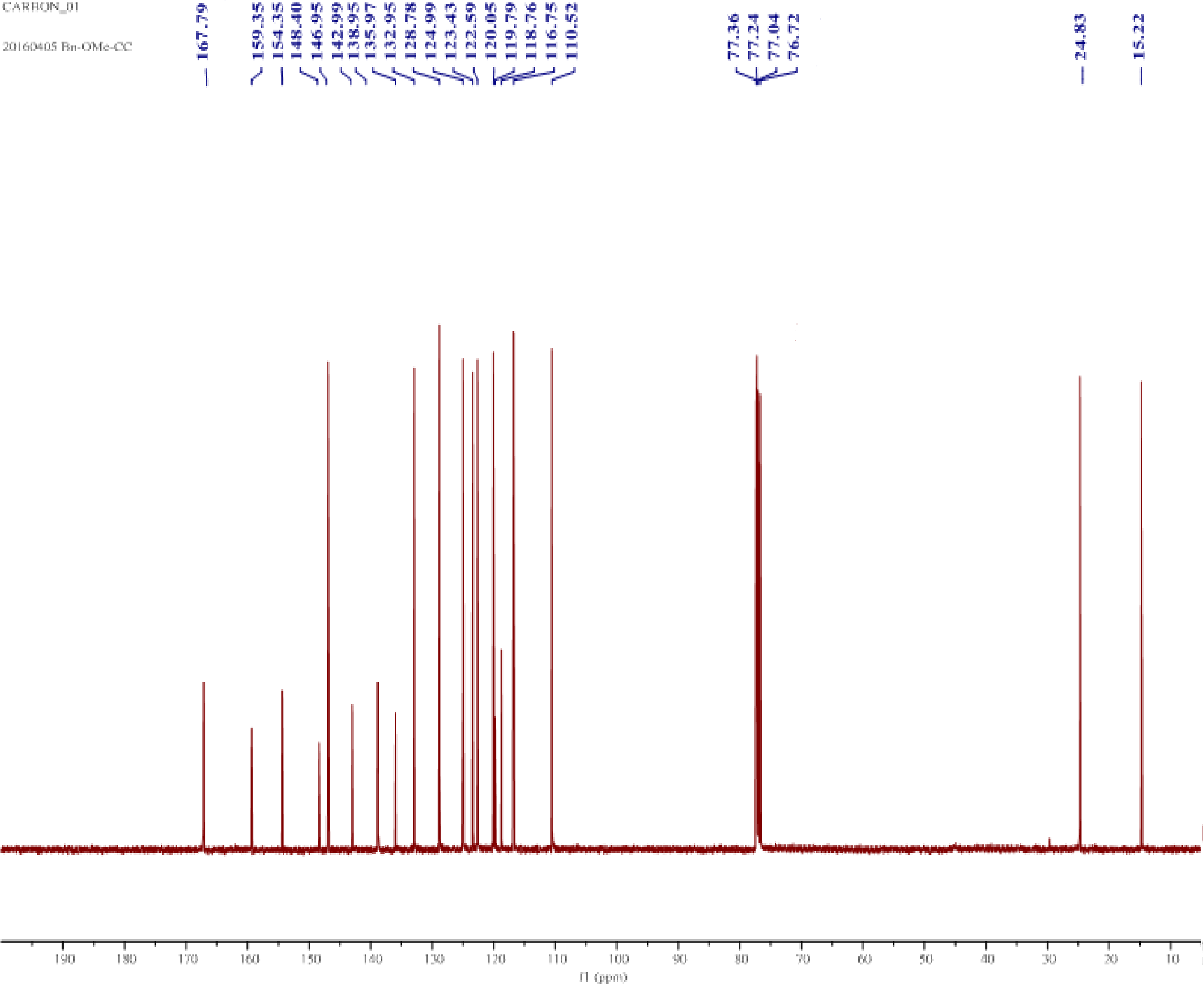

Supplement: S13 Fig — (TIF) [file pone.0196016.s013.tif]

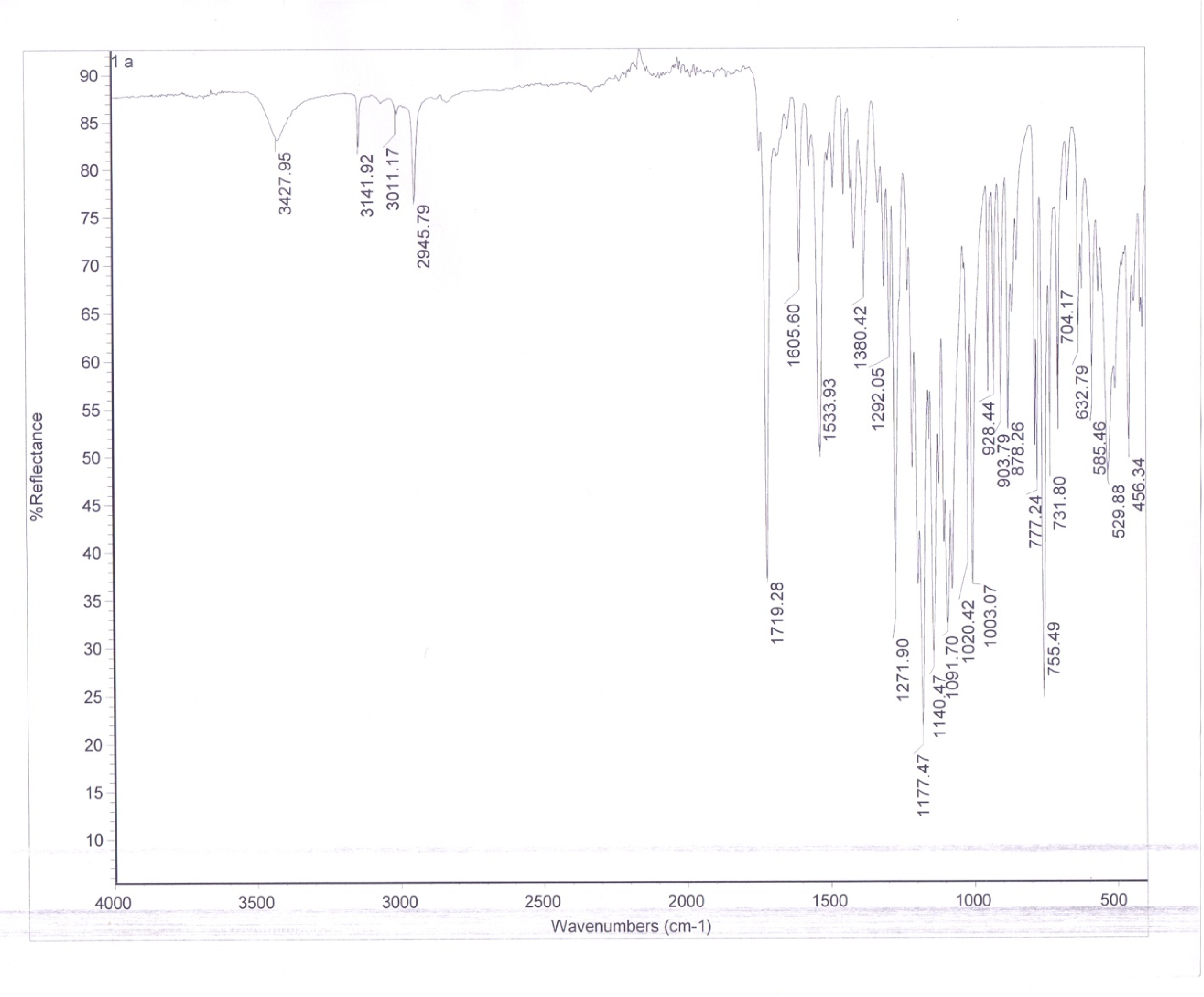

Supplement: S14 Fig — (TIF) [file pone.0196016.s014.tif]

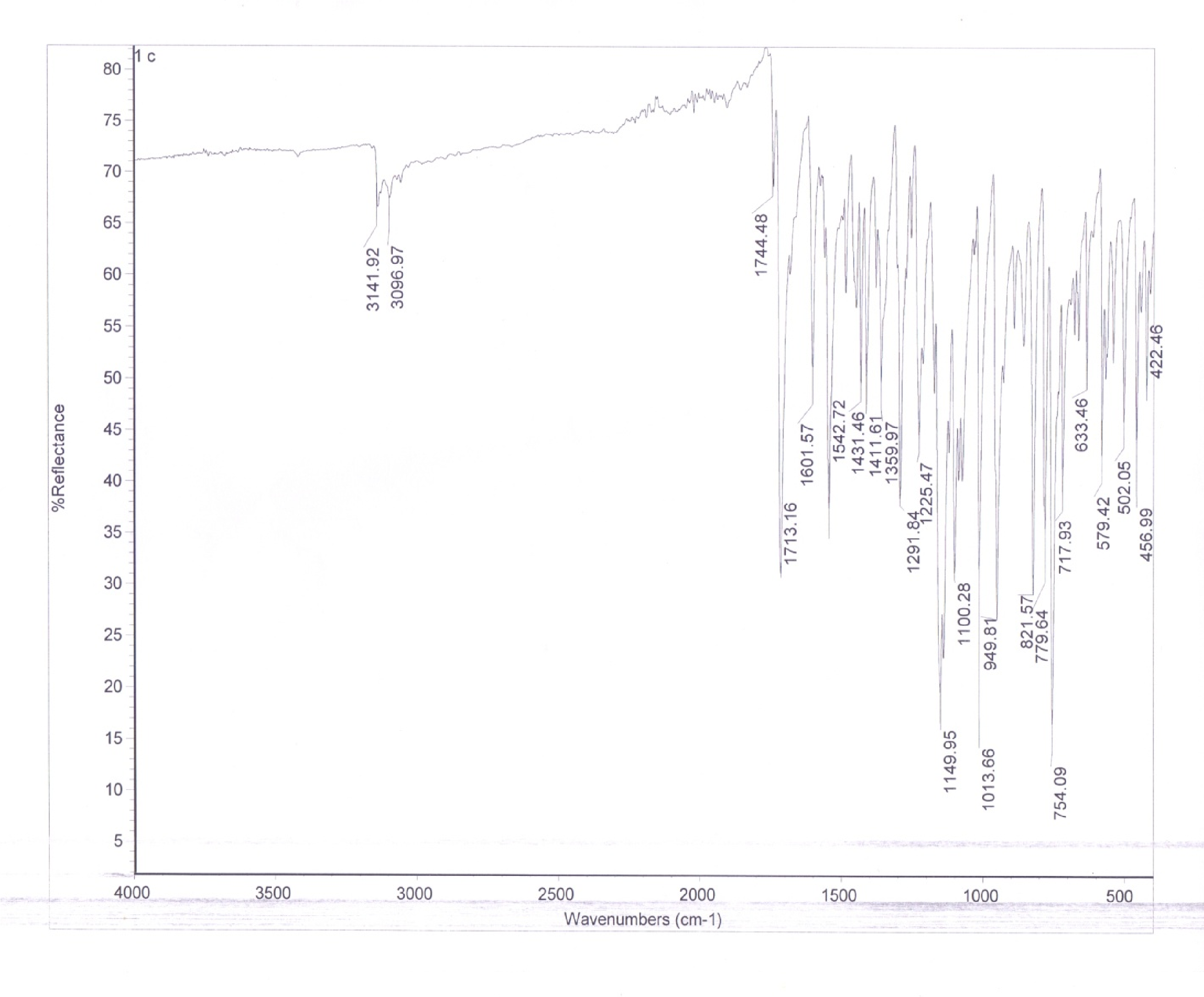

Supplement: S15 Fig — (TIF) [file pone.0196016.s015.tif]

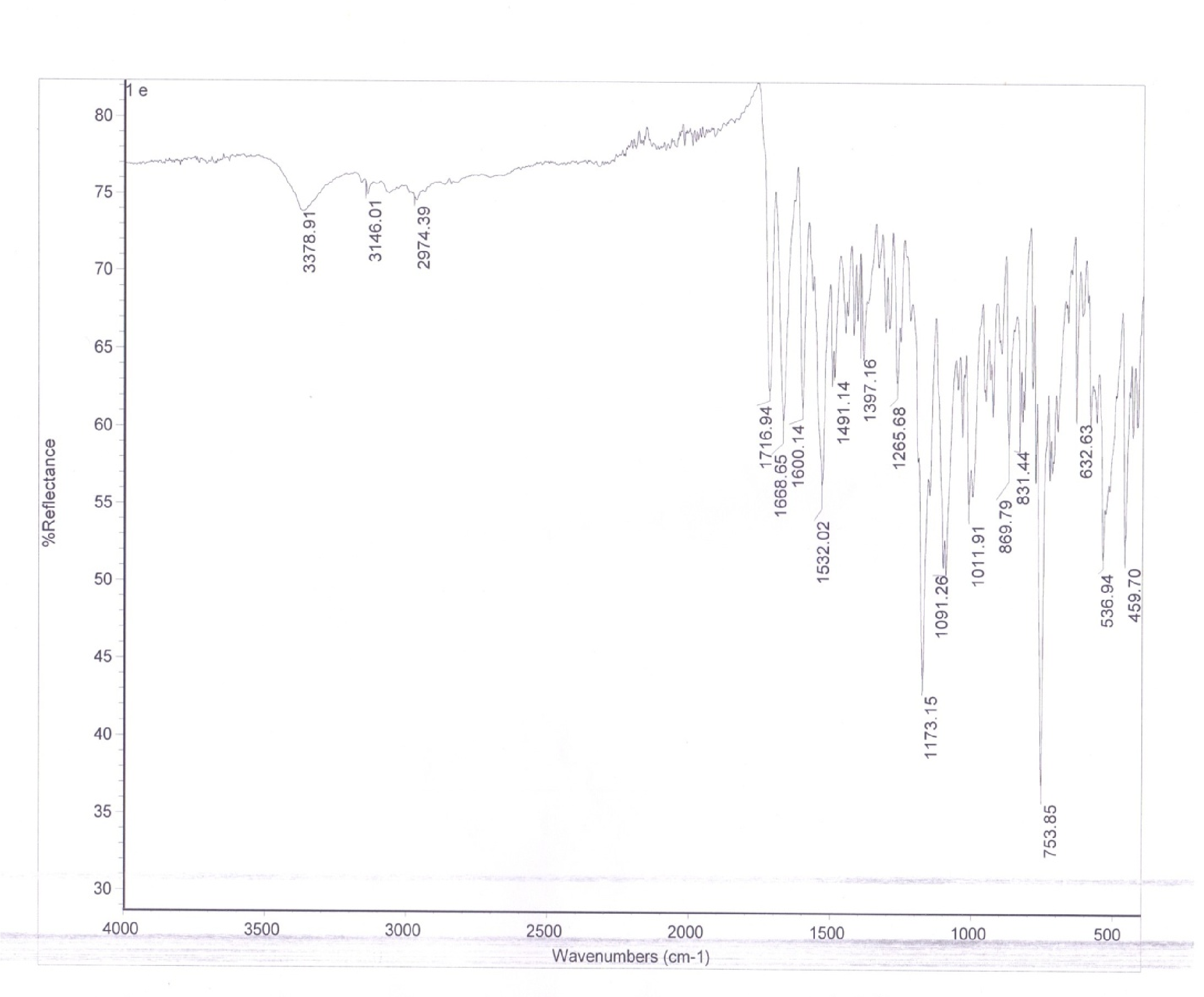

Supplement: S16 Fig — (TIF) [file pone.0196016.s016.tif]

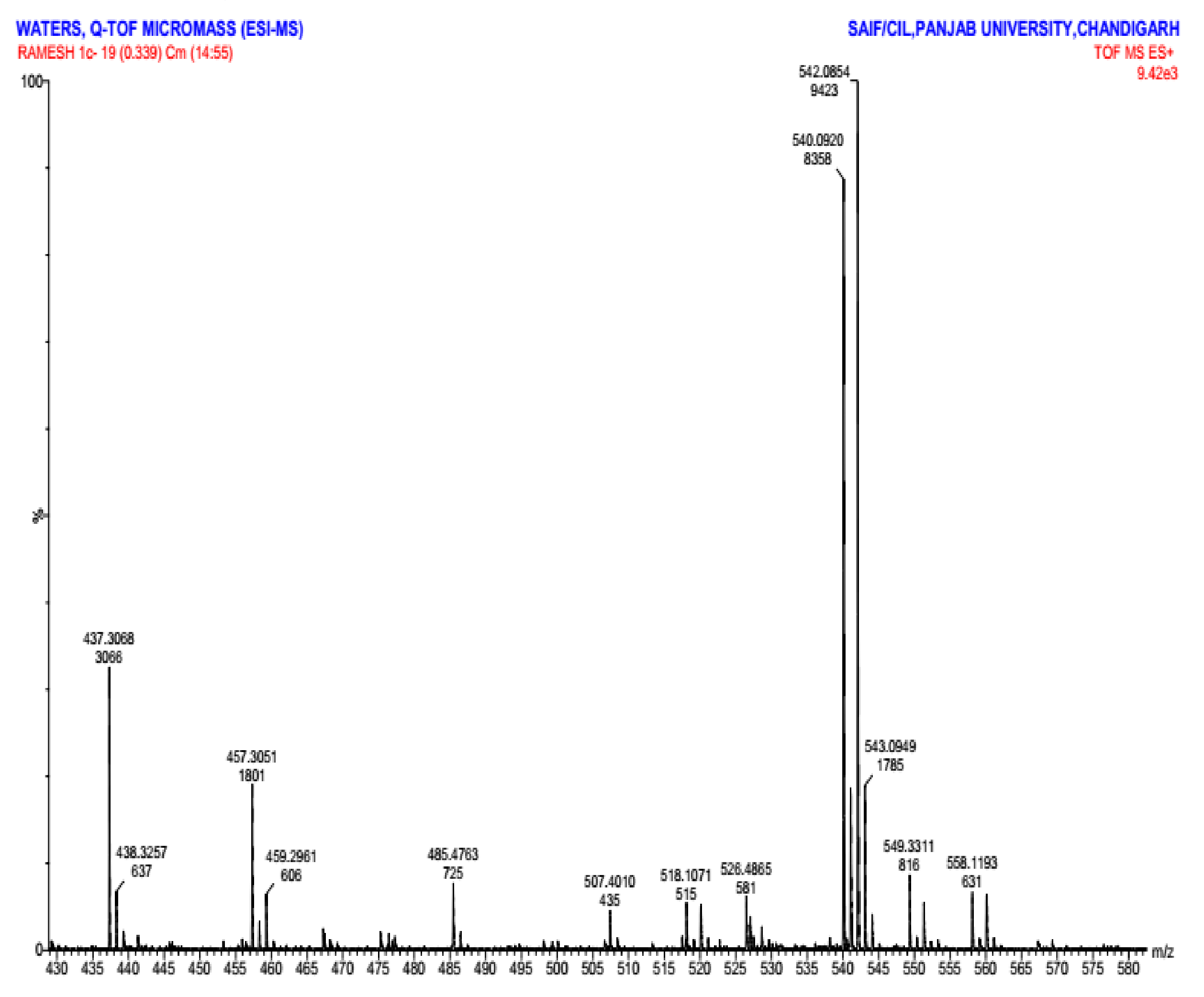

Supplement: S17 Fig — (TIF) [file pone.0196016.s017.tif]

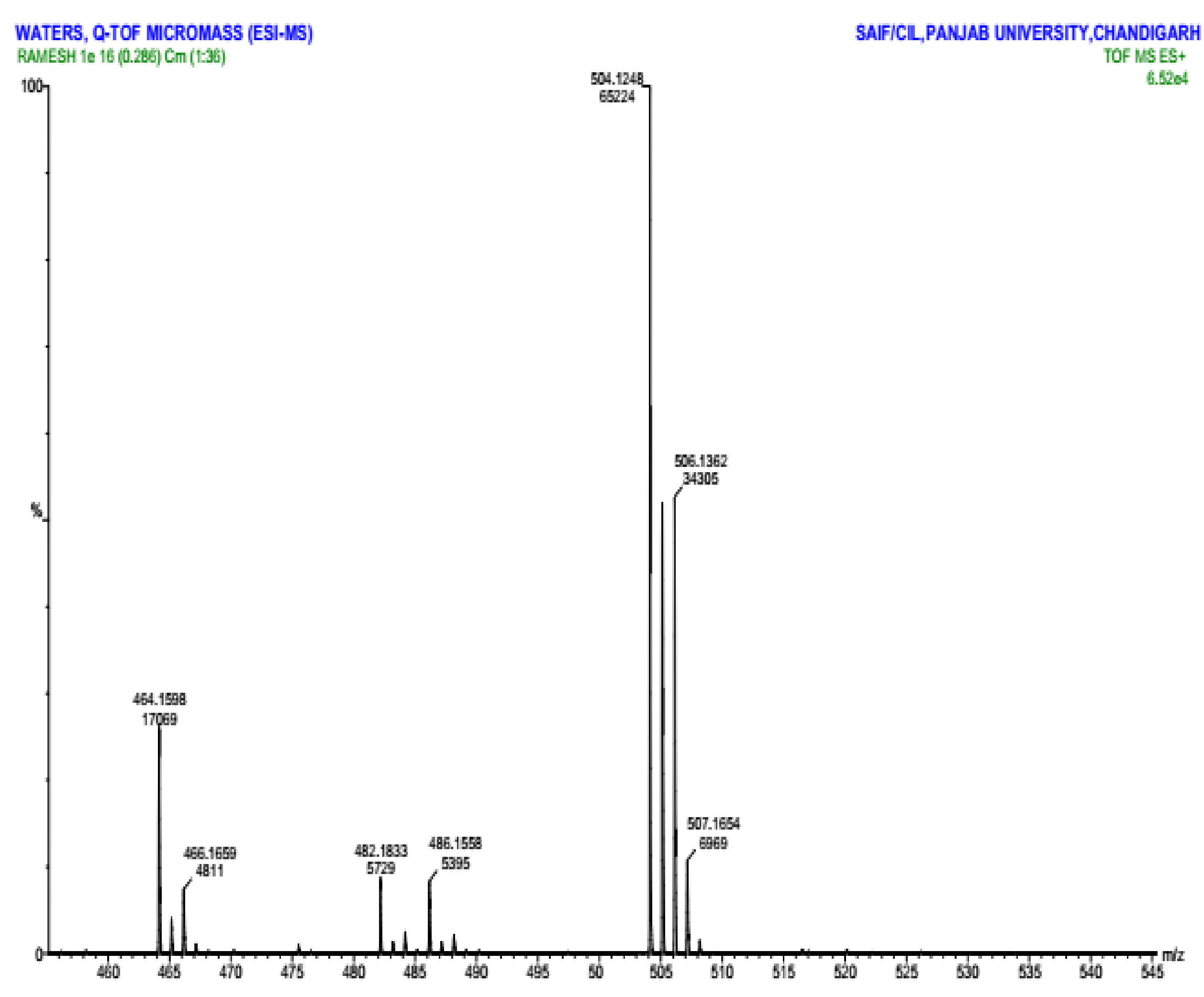

Supplement: S18 Fig — (TIF) [file pone.0196016.s018.tif]

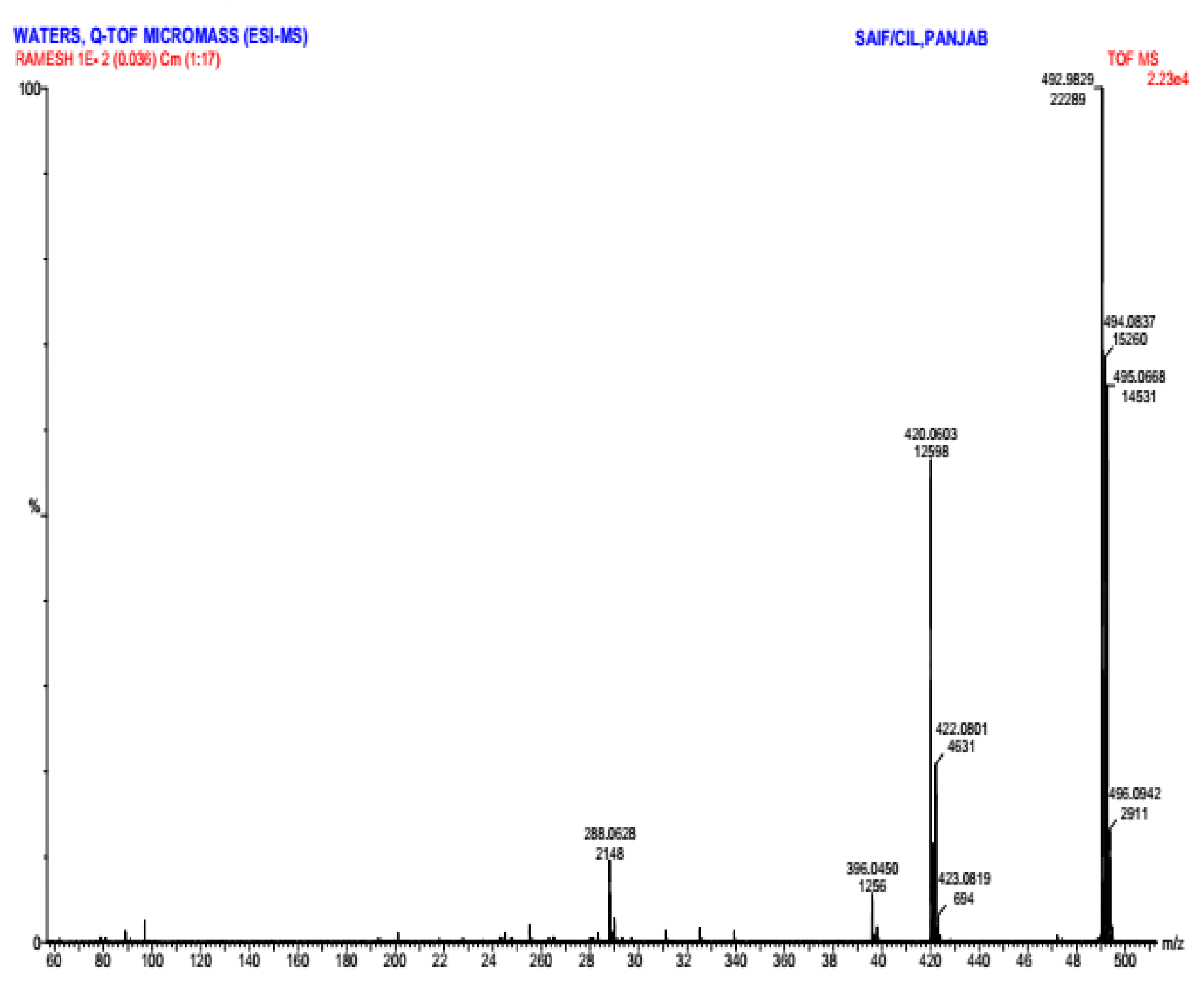

Supplement: S19 Fig — (TIF) [file pone.0196016.s019.tif]
